# Programs and practices that support pregnant people who use drugs’ access to sexual and reproductive health care in Canada: a scoping review

**DOI:** 10.1186/s12884-023-06225-w

**Published:** 2024-01-22

**Authors:** Holly Mathias, Lesley Ann Foster, Ashleigh Rushton

**Affiliations:** 1 School of Public Health, University of Alberta, 11405 87 Ave NW, Edmonton, AB T6G 1C9 Canada; 2 Department of Cultural Studies, Queen’s University, 99 University Ave, Kingston, ON K7L 3N6 Canada; 3 Faculty of Health Sciences, The University of the Fraser Valley, 45190 Caen Ave, Chilliwack, B.C V2R 0N3 Canada

**Keywords:** Harm reduction, Health services, Reproductive health, Reproductive justice, Substance use

## Abstract

**Background:**

Pregnant people who use unregulated drugs (PPWUD) are at high risk of health complications yet experience a range of barriers to sexual and reproductive health care. Given that improving maternal health and access to reproductive health care are key targets underpinning the Sustainable Development Goals (SDG), there is an urgent need to improve access to appropriate supports and services for this population. Little is known about what programs and practices exist to support PPWUD’s access to sexual and reproductive health care. This scoping review aimed to identify the available literature on these programs and practices in Canada.

**Methods:**

A scoping review was conducted using JBI methodology and reported using PRISMA guidelines. Scholarly databases and grey literature sources were searched to identify literature published between 2016–2023 in English or French that discussed, defined, conceptualised, or evaluated programs and practices that support PPWUD’s access to sexual and reproductive health care in Canada. Identified literature was screened using Covidence. Data were extracted from included texts, then analysed descriptively. Frequencies and key concepts were reported.

**Results:**

A total of 71 articles were included, most of which were grey literature. Of the total, 46 unique programs were identified, as well as several useful practices. Most programs were in urban centres in Western Canada, and most programs offered holistic ‘wrap-around services.’ Several programs delivered these services on-site or as ‘drop-in’ programs with the support of staff with lived/living experience of substance use. Most frequent program outcomes included keeping parents and children together, improving connection to other services, and reducing substance use harms. Noted helpful practices included non-judgmental care and the use of harm-reduction strategies.

**Conclusions:**

Several programs and practices that support PPWUD exist in Canada, though few focus exclusively on sexual and reproductive health. There remain opportunities to improve access to programs, including expanding geographic availability and range of services. The review has clinical application by providing an overview of available programs that may support clinicians in identifying services for PPWUD. Future research should consider client perspectives and experiences of these programs.

**Review registration number:**

Open Science Framework https://osf.io/5y64j.

**Supplementary Information:**

The online version contains supplementary material available at 10.1186/s12884-023-06225-w.

## Background

Globally, more than 54.4 million women^1^ use illicit or unregulated drugs (e.g., synthetic opioids, methamphetamines, crack cocaine) [1]. Many women are of childbearing age and may experience pregnancy and substance use simultaneously [2]. Using unregulated drugs during pregnancy is an urgent public health concern given the ongoing global unregulated toxic drug crisis (sometimes referred to as ‘overdose crisis’) [3–5]. It is estimated that 5–6% of people in North America use illicit drugs while pregnant [6]. This number is disproportionately higher among Indigenous people in Canada due to a legacy of colonial violence, systemic racism, and health inequities [7–9]. Although alcohol, tobacco and cannabis use are most reported; there has been an increase in reported opioid use during pregnancy [10–12]. Among people using opioids in the United States, approximately 80% of pregnancies are unintended compared to approximately 45% of the general population [13–15]. The high rate of unplanned pregnancies is attributed to low use of contraceptives, misconceptions about fertility while using drugs, and limited access to sexual and reproductive health (SRH) services [11, 16–19]. People may continue to use drugs throughout their pregnancy for a number of reasons, including to manage withdrawal, or to cope with an unexpected pregnancy, gender-based violence and other traumas [2, 20]. Substance use during pregnancy can create unique harms for the birth parent and foetus. In addition to gendered substance-use related harms (e.g., increased risks of HIV and hepatitis C), pregnancy-related harms include transmission of HIV, low birth weight, neonatal abstinence syndrome, preterm delivery, maternal morbidity, placenta rupture, and delayed child development [11–14, 16, 17].

Many PPWUD want access to SRH services, but there is an unmet need and deep inequities in access [16, 17, 21–23]. SRH services focus on promoting and maintaining good sexual health through information and education, access to family planning, preventing, and managing sexually transmitted infections (STIs), and supporting healthy pregnancies [24]. Key barriers to accessing SRH services include fear of forced abortion/sterilisation, fear of their child(ren) being taken into state care due to parental substance use, disapproval from partner, stigma, fear of judgement, lack of knowledge of service options, limited service availability, limited transportation, limited hours of operation, mistrust of health services, negative previous experiences and cost of uninsured services [11, 13, 14, 17, 22, 25–28].

Despite these barriers, there are some promising practices that support PPWUD’s access to SRH services. In the United States, the integration of SRH services in primary care, substance use treatment and harm reduction programs have supported access to contraception and family planning services [11, 29, 30]. Other research has suggested that PPWUD prefer to access SRH services in spaces they already frequent, such as harm reduction programs, or through mobile outreach services [15, 31, 32]. Additional facilitators of access include strong social support networks, trusting relationships with medical providers, gender-specific services (e.g., counselling for gender-based violence), accessible transportation, no-cost services, walk-in and virtual services, augmented program funding, and dedicated gender and addictions medical training [13, 15, 32–36]. Nevertheless, programs and practices must also transcend the individual and interpersonal levels to address structural and rights-based barriers [37]. Some countries have begun to address structural barriers by providing clear directives and standards of care for PPWUD. The Department of Health and Aged Care in Australia, for instance, has a national Pregnancy Care Guideline that outlines the standards of care that all PPWUD should receive [38]. This has been reflected in specific services for PPWUD across Australia [39].

### Reproductive justice for pregnant people who use drugs

In Canada there continues to be a lack of research that examines how race, class, and gender inequalities relate to social determinants of health and affect reproductive care and bodily autonomy [40, 41]. Increasingly, Canadian professionals and scholars in the field of sexual and reproductive health and rights (SRHR) are calling for “a paradigm shift” that integrates a reproductive justice framework into the field of SRHR, recognizing historical, systemic, and structural violence that (re)produces stigma and contributes to the social determinants of substance use [41, 42]. Moulded by the work of Black women, women of colour and Indigenous feminist activists, reproductive justice goes beyond the reproductive rights framework of privacy, liberal individualism and abortion access to address interconnecting reproductive issues and violations [41, 43]. This paradigm shift has great implications for PPWUD as they have unique reproductive health needs as a high-risk and vulnerable population, including prenatal care, harm reduction, culturally-based practices for rehabilitation, emotional support, housing and beyond [44]. The right to parent in healthy communities, “free from violence by individuals or the state” is an important aspect of reproductive justice, and speaks to the needs of PPWUD population, through an intersectional and trauma-informed lens [43].

While this paper includes all PPWUD in Canada, it is crucial to recognize that Indigenous peoples have been largely absent from research, data, policies, and programs on PPWUD in Canada [42, 45]. When examining reproductive justice and PPWUD in Canada, it is crucial to consider colonialism, racism, and multiple forms of violence that Indigenous people experience [41]. Drawing on the work of Dell and Lyon (2007), Allen explains that the Indigenous population in Canada, including First Nations, Métis, and Inuit peoples, is the second largest Indigenous population in the world proportional to the general population [46]. Among Indigenous populations in Canada, substance use is one of the most acute social and health issues [7, 42, 46]. Through incorporating an intersectional and reproductive justice framework, SRH services can provide strategies and holistic care for Indigenous communities impacted by violent colonial legacies. Promisingly, Canada has embraced the use of the Sustainable Development Goals (SDGs) framework and has committed to improving maternal health outcomes as a priority [47].

### Positioning pregnant people who use drugs within the sustainable development goals

Adopted at the United Nations (UN) in 2015, the SDGs set out targets and indicators across 17 goals to achieve “peace and prosperity for people and the planet, now and into the future” [48]. As McArthur & Rasmussen (2019) contend, the overall aim of the SDGs should be to “leave no one behind,” which requires ensuring the most vulnerable and marginalised people are included in strategies to achieve sustainable social, economic, and environmental wellbeing [49]. Specific focus on the intersections of pregnancy, drug use and access to SRH care is limited within the SDG agenda. However, it is valuable to examine the goals and targets which are of relevance to PPWUD when accessing healthcare services to understand how the targets can better support this population (see Additional file 1).

The 2022–2026 Federal Sustainable Development Strategy (FSDS) provides a domestic strategy to Canada’s commitment to working towards achieving all 17 SDGs [50]. Within the strategy, inequities within and across Canadian populations are acknowledged, including unique challenges experienced by Indigenous Peoples, members of the 2SLGBTQIA + community (Two-Spirit, Lesbian, Gay, Bisexual, Transgender, Queer or Questioning, Intersex, Asexual and additional sexual orientations and gender identities), youth, and ethnic minority people. However, there is an absence of commitments to work towards the targets relevant for PPWUD. This raises questions on whether PPWUD will fall through the gaps and if specific funding will be made available for tailored support.

Unlike some developed nations, such as Australia and Germany, Canada is on track to meet SDG goal 1: Ending Poverty [51]. Reflecting on similar progress in other developed countries such as the United Kingdom (UK) and United States of America (USA), Canada has met target standards for reducing neonatal and maternal mortality [51, 52]. However, The United Nations state that there is slow progress in achieving worldwide gender equality, as many countries, including Canada, UK, and USA are failing to reach goal 5 and target 3.7 (goal 3) on universal access to reproductive health services [51–53]. While it is reassuring to note some of Canada’s progress in achieving the SDGs in comparison to other developed countries, challenges remain for Canada to meet key targets. Drawing on the SDGs offers a clear pathway to increase the wellbeing of marginalised populations. By providing tailored support for PPWUD, Canada can actively work towards ensuring the identified targets are met.

Existing research on PPWUD has focused on access to contraceptives and HIV testing [11, 18], access to substance use treatment and harm reduction [32, 54, 55], and barriers to accessing SRH services [13, 32]. There is limited evidence regarding promising practices that support PPWUD’s access to SRH services, particularly in Canada [56]. The research that does exist primarily focuses on alcohol, tobacco and cannabis use which are regulated legal substances in Canada. There remains a knowledge gap on opioid and polysubstance use, especially amidst the deadly toxic drug crisis [56, 57]. This crisis has claimed the lives of over 38,000 Canadians since 2016, with thousands more experiencing non-fatal, but life-changing health impacts, including brain injury. The serious harms attributed to this crisis have resulted in increased government investment through the Canadian Drugs and Substances Strategy, as well as a declaration of a state of emergency by several provinces and territories, and Treaty 6 Confederacy representing First Nations people in Alberta [3–5]. The urgency of this public health issue underscores the need to better understand how to support all people who use unregulated drugs, including PPWUD [57].

The SDGs and Canada’s commitment to improving maternal health outcomes in the context of reproductive justice underscores the importance of including marginalised populations to ensure no one is left behind. Grounded in the values of reproductive justice and sustainable development, a scoping review was conducted to 1) explore the existing subject literature and 2) provide an overview of programs and practices that support PPWUD’s access to SRH services. The intent is that this review will provide insight into current programs and practices in Canada, and map areas for future research, clinical and program development. Specifically, we asked what existing programs and practices support PPWUD’s access to SRH services in Canada? What are the key components of these programs and practices?

## Methods

We conducted a scoping review using Joanna Briggs Institute (JBI) methodology and reported using PRISMA-ScRV [58, 59]. Our protocol is registered with Open Science Framework (https://osf.io/5y64j). Before conducting the review, we systematically searched for other synthesis articles and protocols through Cochrane Systematic Reviews and JBI. No other reviews on this topic were found.

### Search strategy

The search strategy aimed to identify both peer-reviewed and grey literature, including published and unpublished primary studies, reviews, text and opinion papers, systematic reviews, dissertation and theses, commentaries, media articles, websites, full conference presentations and reports. We focused on literature on programs and practices that provide access to SRH and related care for PPWUD in Canada at a municipal, provincial/territorial, or federal level.

A list of search terms was developed by the authors who have expertise in substance use, SRH, and women’s and allied health. An initial search on MEDLINE (OVID) in March 2023 identified relevant articles and other additional search terms. Based on these articles and terms, a full search strategy was developed and tested with a health sciences librarian (see Additional file 2). During our initial search, we noted that many research articles on SRH do not explicitly refer to “sexual and reproductive health” in the text. For example, some research on contraceptive use among women who use drugs used the term “contraception” and not “sexual and reproductive health”. To ensure a comprehensive search capturing a broad range of programs, in line with a scoping review approach, we chose to omit the specific term "sexual and reproductive health" from the search strategy. Specific services within this domain would be identified during the screening and extraction stages of the review. Additionally, we included terms referring to various stages of the pregnancy journey to capture services that may be offered at different points in time (e.g. prenatal support and postpartum programs).

The term ‘drugs’ was defined based on Health Canada’s definition of illicit drugs [60]. We defined programs as being population-level or individual-level programs with any length of delivery (e.g., short-term, or long-term) provided by a recognized group or institution (i.e., any level of government, private sector, or non-profit). We included articles published between 2016 and 2023 in English and French. This period was selected to capture the height of the toxic drug crisis in Canada. Frequency of drug use and age were not restricted in the search. Ineligible texts included conference abstracts, letters, meeting minutes, blog posts, speeches and/or transcripts from legislative assemblies. Any peer-reviewed article not available as full-text through institutional library holdings from University of Alberta, Queen’s University and the University of the Fraser Valley were excluded.

### Evidence sources

**Fig. 1:**
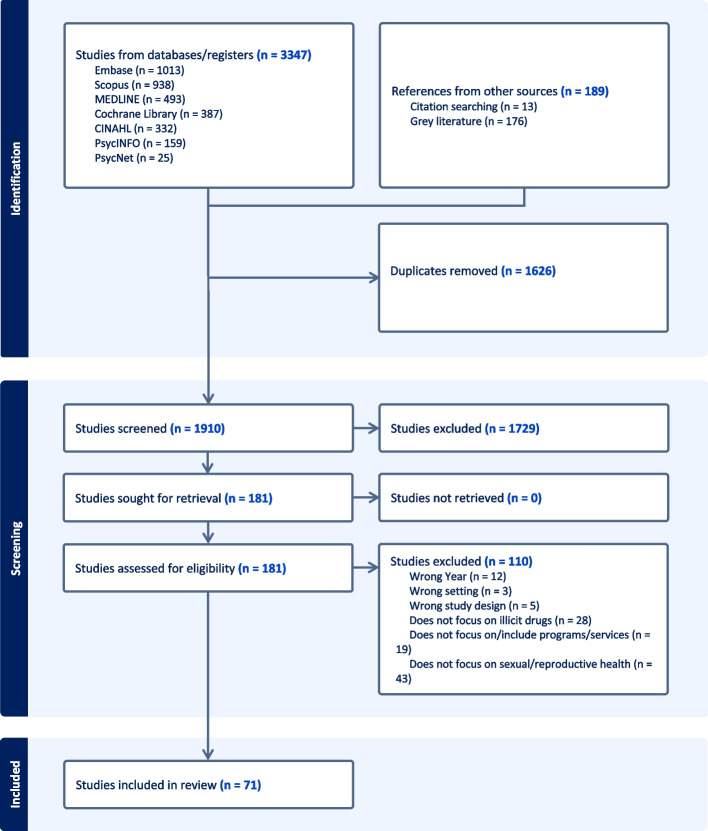
PRISMA flow diagram of the scoping review process including number of texts identified, number of texts excluded with justification and final number of included texts

We searched MEDLINE (Ovid), EMBASE (Ovid), PsychINFO (Ovid), PsychNET (OVID), CINAHL, Scopus, Cochrane Library, and Google Scholar. Grey literature was identified using the first five pages of Google search results, as well as a targeted search of relevant Canadian harm reduction organisations, multi-service programs, and provincial/territorial health authorities. All identified records were collated and uploaded into Covidence (Veritas Health Innovation, Melbourne, Australia) with duplicates removed. Following a pilot test of five peer-reviewed articles, titles and abstracts of peer-reviewed texts were screened by two independent reviewers against the inclusion criteria. Any conflicts were resolved through discussion to reach consensus. Grey literature was reviewed by three reviewers against the inclusion criteria. After initial screening, the full text of each peer-reviewed and grey literature article was reviewed by two independent reviewers with disagreements resolved through consensus. A total of 3347 texts were identified from our search and after duplicates were excluded (*n* = 1626), 1910 titles and abstracts were screened (see Fig. 1) [61]. From this initial screening, 1729 studies were excluded, and the remaining 181 full texts were screened. A further 110 studies were excluded and a total of 71 texts were included in the review.

### Data extraction

A data extraction tool was developed and piloted by the authors. The data extraction tool included specific details about the article, population, programs or practices, context, and key findings. The tool was piloted by three independent reviewers by extracting data from four articles and comparing codes. Data were extracted from included texts by two independent reviewers and any conflicts were resolved through discussion.

Key concepts were developed by reading and re-reading the data individually, and then discussing potential concepts as a group. Data were then read and coded inductively. Codes were grouped together to form key concepts. Please note that some of the articles reported information on multiple programs, so we have reported frequencies based on the total number of programs reported rather than number of included articles.

We noted that many programs provided SRH-adjacent services for pregnant people but did not explicitly refer to “sexual and reproductive healthcare”. For example, some articles referred to providing primary care for PPWUD throughout pregnancy. Although this care supported their reproductive health, it was not explicitly labelled as such, thus we included this service under “physical health”. Due to few services/programs explicitly labelled as being SRH services, we decided to report on all programs that informed the SRH and wellbeing of parent and baby.

## Findings

**Fig. 2:**
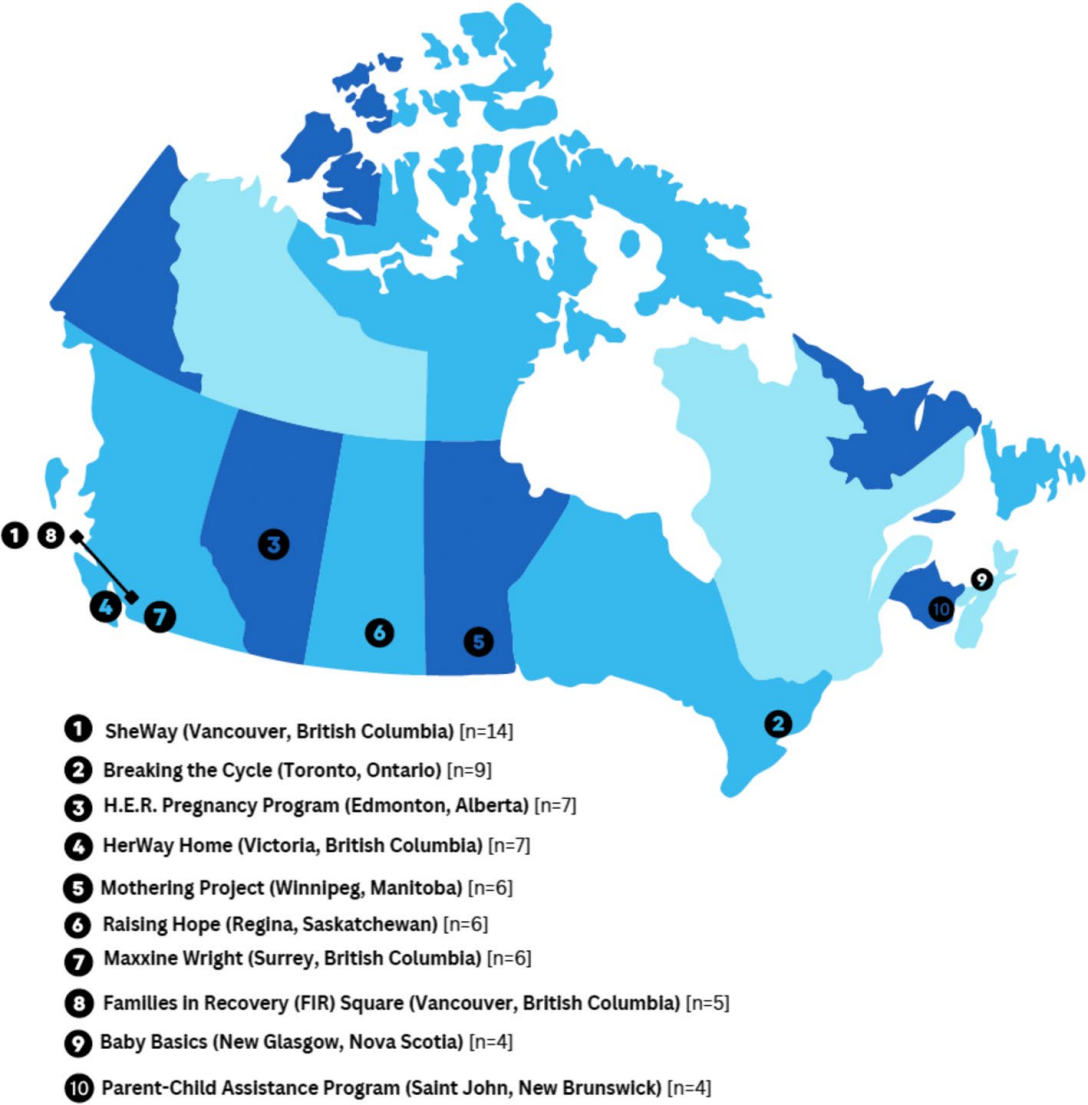
Map of the top 10 most frequently identified programs in Canada for PPWUD as identified in our review. The frequency of times mentioned in the included texts is indicated in square brackets after the program name and location

Of the 71 included articles, a total of 15 articles (21.1%) were peer-reviewed and 56 (78.9%) were grey literature, including reports, websites, white papers or policy papers, conference presentations and media articles. As many articles discuss multiple programs with multiple articles discussing the same programs and services, we identified 46 unique programs across the 71 included articles (see Table 1 and Fig. 2). The objectives of the included articles were to: provide information about the programs offered (*n* = 45) (for example see [62, 63]), present findings from research evaluations or analysis (*n* = 19) (for example see [64, 65]) or offer a blueprint or toolkit for service providers (*n* = 3) [66, 67]. The purposes of four articles were unclear. Not all programs had strict eligibility criteria on type of substance use. For example, 19 articles reported that the programs assist pregnant people who experience drug and alcohol use disorders (for example see [68, 69]) and 13 articles noted that support is given to pregnant people who have problematic substance use (for example see [70, 71]). Specific mention was given to opioid use (*n* = 11) (for example see [72–74]), illicit drug use (*n* = 7) (for example see [75, 76]), with one article of the seven articles referring to ‘crack and meth’ [77]. Furthermore, the general term ‘drugs’ was used within four articles with no specific drug noted.

**Table 1:** Number of identified programs and drug trends by province/territory (2023)^a^

Province/Territory	Number of Identified Programs	Apparent opioid toxicity deaths per 100,000 (January-March 2023) [3]	Opioid-related poisoning hospitalizations per 100,000 (January-March 2023) [3]	Stimulant-related poisoning hospitalizations per 100,000 (January-March 2023) [3]
Canada	1 (Federal)	20.3	17.4	6.4
British Columbia	15	48.1	26.1	7.1
Alberta	8	38.2	25.3	7.4
Saskatchewan	2	13.1	19.1	12.1
Manitoba	2	N/A	4.8	2.6
Ontario	10	17.0	14.3	6.0
Quebec	2	5.1	N/A	N/A
New Brunswick	2	5.4	9.9	6.4
Nova Scotia	2	5.9	7.1	3.9
Prince Edward Island	0	4.7	S	S
Newfoundland and Labrador	0	6.8	12.9	4.6
Yukon	0	27.4	21.6^a^	S
Northwest Territories	0	0.0	21.6^a^	S
Nunavut	0	0.0	21.6^a^	S

The number of unique programs identified in our review and trends in drug-related deaths and hospitalizations per 100,000 (January-March 2023) reported by province and territory

*N/A* Data not available

S Data suppressed due to low numbers in order to comply with confidentiality rules

^a^Data for all three territories combined

### Program context

Across the 71 articles analysed, specific information was provided regarding program context, including name of interventions, location, funding source, target population, and purpose of the program’s intervention. We identified 46 unique programs and services for PPWUD in Canada (*n* = 46). Although there were many diverse programs available, large areas of the country were without access to vital health services for PPWUD (see Table 2 for program details).

**Table 2:** List of unique programs and services for PPWUD in Canada

Program Name	Location	Population	Year Founded	Purpose	Type of Program
Provincial Advocacy Network of Women Supporting Women [66]	British Columbia (provincial)	5,000,879	N/A	Provides peer support for women and people who use substances, including coordinated efforts to address harmful policy and practices that affect the women in the network and the people they represent	Non-profit
Provincial Healthy Care Pregnancy Program [66, 78–80]	British Columbia (provincial)	5,000,879	N/A	Aims to better support pregnant, postpartum and newly parenting people who use or have used substances. It creates and utilises formal connections between acute care facilities and community organisations	Non-profit/Registered charity
Pregnancy Outreach Program [81]	Abbotsford, British Columbia	153,524	N/A	Supports women throughout their pregnancies until six months after delivery, by promoting positive health practices and adequate weight gain for both the mother and child	Non-profit
Transition to New Beginnings [82]	Burnaby, British Columbia	249,125	N/A	Provides transitional housing in a semi-independent living environment for pregnant women or new moms with infants, with low substance use needs	Non-profit
The Tree [79, 83]	Kamloops, British Columbia	97,902	N/A	Supports women’s belonging and recovery by walking with women and their families, facilitating healing, connections, and support	Non-profit
Baby’s Best Chance Pregnancy Outreach Program [81]	Mission, British Columbia	41,519	N/A	Supports women who are experiencing high-risk pregnancies, teens, women with drug or alcohol concerns, and women with low income	Non-profit
Karis Support Society [84]	Okanagan, British Columbia	403,940	2008	Contributes to genuine social transformation in the Okanagan Valley by providing a safe home, support through recovery and life skills development for people struggling with life altering additions and mental health conditions	Non-profit
Harmony House [85]	Prince George, British Columbia	76,708	N/A	Supportive housing for women who are struggling with mental health and/or problematic substance use, are pregnant or new mothers in danger of losing their child to the Care of the Ministry of Children and Families Development	Non-profit
Ellendale Cradle [82]	Surrey, British Columbia	568,322	N/A	Helps women break the cycle of poverty, addiction, mental illness, homelessness, and crime	Non-profit
Maxxine Wright Community Health Centre [64, 65, 86, 87]	Surrey, British Columbia	568,322	2005	Supports women who are pregnant or who have very young children at the time of intake who are also impacted by substance use and/or violence and abuse	Non-profit
Circle of Birth Keepers [66]	Surrey, British Columbia	568,322	N/A	Actively addresses the needs and gaps in birth work by training more Indigenous birth keepers and providing access to wrap-around care for Indigenous birthers to access support, programs, services, information, and supplies	Non-profit
Families in Recovery (FIR) Square [64, 66, 68, 69, 72, 88, 89]	Vancouver, British Columbia	662,248	2003	The first program in Canada to care for women who use substances and their newborns exposed to substances in a single unit; helps women and their newborns stabilise and withdraw from substances, keeping mothers and babies together whenever possible and provides care from antepartum to postpartum and between hospital and community	Provincial health authority
SheWay [64, 65, 89–99]	Vancouver, British Columbia	662,248	1993	Offers a broad range of on-site health, medical and social services and supports for women who are either pregnant or parenting young children and who are experiencing current or previous issues with substance use	Non-profit
HerWay Home [65, 76, 90, 98–101]	Victoria, British Columbia	91,867	2013	Provides non-judgmental health care and social supports for pregnant and parenting women who have a history of substance use and may also be affected by mental health issues, violence, and trauma	Provincial health authority
Mothers for Recovery [102]	Victoria, British Columbia	91,867	N/A	Supports all pregnant, postpartum, and parenting people who identify as mothers and have or are using substances	Non-profit
Parent–Child Assistance Program (PCAP) [67, 103, 104]	Alberta (Provincial)	4,262,635	N/A	Supports women to reduce or stop alcohol and/or drug use during pregnancy, to achieve and maintain recovery, and to support healthy pregnancies and lives for women and their children by addressing the needs of mothers and getting them stabilised in a whole host of ways	Provincial government
Aventa Centre of Excellence for Women with Addiction [62, 105]	Calgary, Alberta	1,306,784	1970	Offers a holistic treatment approach in helping women to overcome their addictions, and lead lives that are addiction free. Women help women to build resilience so that they may overcome the effects of trauma and addictions	Private with some provincially-funded services
Rapid Access Addiction Medicine Clinic [72]	Calgary, Alberta	1,306,784	N/A	Alberta Health Services physicians are working to launch a “rooming-in” program. The service would allow mothers and babies to stay together with a range of supports, related to withdrawal or stabilisation from drug use, parenting techniques and specialised care for newborns	Provincial health authority
H.E.R. Pregnancy Program [64, 65, 86, 90, 98, 106]	Edmonton, Alberta	1,010,899	2011	Works to decrease barriers to health and social services for street involved pregnant women by firmly grounding their work in a harm reduction approach and by providing culturally safe and trauma-informed care	Non-profit
Health for Two [107]	Edmonton, Alberta	1,010,899	N/A	A free program for women who need extra support to have a healthy pregnancy. Health for Two provides support throughout pregnancy and up to 2 months postpartum to women who require extra support to have a healthy pregnancy	Provincial government
Aboriginal Prenatal Wellness Program [97]	Maskwacis First Nation, Alberta	64	2005	A culturally safe program that provides client‐centred prenatal care that is designed to empower women, families and communities. It was created in 2005 to serve Aboriginal women who weren't accessing the traditional system for prenatal care	Non-profit
EMBRACE [71, 73, 74]	Red Deer, Alberta	100,844	2019	Brings together community partners and a multidisciplinary healthcare team to teach new moms who have used opioids during pregnancy how to care for their newborns — free of stigma and shame	Provincial health authority
The Women’s Program [108]	Red Deer, Alberta	100,844	N/A	Addresses the holistic needs of each woman, often facilitating connections to community and medical support and services. This includes: -Pregnancy -Personal health -Housing -Family and legal support -Addiction -Mental health	Non-profit
Prenatal Outreach and Resource Team [109]	Regina, Saskatchewan	226,404	2021	The program is designed to create a culture shift in Saskatchewan that wraps supports around pregnant women with complex life situations by providing early and effective interventions in pregnancy that will reduce health complications in mothers and their infants and prevent children from being apprehended at birth	Non-profit/Charity
Raising Hope [64, 65, 86, 90, 98, 99, 110]	Regina, Saskatchewan	226,404	N/A	Offers 24/7 residential support and programming for women who have an active addiction and are pregnant or who have recently had a baby, including supported housing, pre-natal and parenting supports and assistance with addressing addictions	Non-profit
Insight Program [67, 111–114]	Manitoba (provincial)	1,342,153	N/A	An outreach mentoring program that focuses on long term one-to-one relationship building with women and their families for up to three years	Provincial government
Mothering Project [64, 65, 86, 90, 98, 99, 111]	Winnipeg, Manitoba	749,607	2013	Provides support and resources to women who are pregnant or who have given birth in the past 12 months, and are involved with substances. The project helps to provide vital resources including access to support groups, prenatal care, advocacy, and cultural opportunities	Community health center
Grace Haven Young Parent Resource Centre [115]	Hamilton, Ontario	569,353	N/A	Community-based supports for pregnant adolescents/women and young, single parents (mothers and fathers)	Religious organisation
New Choices [64, 115]	Hamilton, Ontario	569,353	N/A	A community day treatment program for women who are pregnant or mothering young children and have substance addictions. Women attend once a week with their children; provides the opportunity to access addiction and parenting services based on individual needs	Religious organisation
Motherwise [63]	Kingston, Ontario	132,485	N/A	Offers information, support, referrals and counselling in a non-judgmental way that enables participants to feel safe and share freely; respects individuals’ goals within a harm reduction framework. You do not need to have custody of your children to receive support	Provincial government
Thrive [116]	Kingston, Ontario	132,485	2017	A program for women who are pregnant and/or parenting children (under the age of 6) who are/have been experiencing a problem with opioids (oxycodone, Percocet®, heroin, Dilaudid®, morphine) or receiving methadone treatment; offers counselling, in-hospital and in-home support visits, parenting support and education, coordinating family care plans, and opportunities for community advisory group participation	Provincial health authority
Oracle Pathway [117]	Ottawa, Ontario	1,017,449	N/A	Provides holistic perinatal care for pregnant people who use substances, with a harm reduction approach; facilitate collaboration between addiction treatment services, community-based antenatal care, outreach nursing, midwifery, and specialist obstetric services	Hospital
Pregnancy/Parenting Outreach Program [118]	Sudbury, Ontario	166,004	N/A	A harm reduction program for pregnant or parenting mothers or fathers with children 6 years old and younger	Provincial government
Breaking the Cycle [64, 65, 90, 95, 98, 99, 119–121],	Toronto, Ontario	2,794,356	N/A	An early prevention and intervention program for pregnant and parenting women using substances and their young children aged 0–6 years	Non-profit
Bridges to Moms [122]	Toronto, Ontario	2,794,356	2013	The program works in a holistic and culturally responsive manner to reduce harm to pregnant and parenting women who use substances. Through this program they are also aiming to improve the lives of their young children	Non-profit
Homeless At-Risk Prenatal [123]	Toronto, Ontario	2,794,356	2007	Provides high‐intensity prenatal home visiting and support to homeless pregnant women in the City of Toronto	Provincial government
Toronto Centre for Substance Use in Pregnancy [124, 125]	Toronto, Ontario	2,794,356	1995	Offers multidisciplinary addiction, obstetric and neonatal care to pregnant women in an empathic and non-judgmental environment	Hospital
Jessie [95]	Montreal, Quebec	1,762,949	N/A	Focuses on engaging parents in behaviour change regarding substance use and improving parenting practices to prevent child welfare reports or eliminate the risk of compromising the child's health and safety	Provincial government
Mains dans la main [95]	Montreal, Quebec	1,762,949	2005	Offers support to future parents struggling with substance abuse, with the aim of maximising their chances of assuming responsibility for their child's care from birth. To this end, the program aims to strengthen and promote collaborative ties that will foster greater consistency in interventions and a better alignment of services between two institutions traditionally called upon to intervene separately in such situations: the hospital environment and the Youth Protection Branch	Hospital
Parent–child assistance program (PCAP) [126]	Saint John, New Brunswick	69,895	2018	Provides personalised support for women/birthing people who use substances by pairing them with an advocate for three years	Non-profit
Strengthening Families Program [126]	Saint John, New Brunswick	69,895	2019	Aims to promote positive relationships and social activities while decreasing the risk of adverse behaviours in the future	Non-profit
Fetal Assessment Treatment Centre [127]	Halifax, Nova Scotia	439,189	N/A	Provides diagnostic services to assess the health or wellbeing of the unborn baby	Provincial health authority
Obstetrical Day Unit [127]	Halifax, Nova Scotia	439,189	N/A	Improves patient care during the antepartum period through a coordinated approach to meet the needs of the patient and her family	Provincial health authority
Prenatal Special Care Unit [127]	Halifax, Nova Scotia	439,189	N/A	Provides care to women and their families experiencing high risk	Provincial health authority
Baby Basics [65, 86, 90, 99]	New Glasgow, Nova Scotia	9,471	1999	Offers a support group at Pictou County Kids First specifically for moms 24 years of age and under	Non-profit
Canada Prenatal Nutrition Program [125]	Federal (Canada wide)	36,991,981	1995	Provides funding to community groups to help to improve the health of pregnant women, new mothers and their babies, who face challenges that put their health at risk, such as: • Poverty • Teen pregnancy • Social and geographic isolation • Substance use • Family violence	Federal government

A list of all unique programs and services that support access to sexual and reproductive health services for pregnant people who use drugs in Canada (as identified in this review); Abbreviations: Parent–Child Assistance Program (*PCAP*), pregnant people who use drugs (*PPWUD*)

In Canada, most programs and services for PPWUD were concentrated in urban centres, leaving large geographical gaps in access to services across suburban, rural and Northern regions of Canada. Most programs were in major metropolitan areas, particularly in Western Canada, including Vancouver, Winnipeg, and Edmonton. Three programs (*n* = 3) [64, 70, 128] did not report a location and no programs were identified in Prince Edward Island, Newfoundland and Labrador, and the three Northern Territories. Most programs and services did not report funding sources (*n* = 59) however, among the articles that did include funding sources, provincial funding (*n* = 30) was most reported, with non-profit organisations second (*n* = 24), followed by federally-funded programs (*n* = 20) and regionally-funded programs (*n* = 14).

The main target population for programs and services was PPWUD (*n* = 44), with some programs overlapping and targeting postpartum and parenting services (*n* = 15) (for example see [66, 78, 86, 88, 102, 103, 121]). Indigenous peoples (*n* = 16) (for example see [65, 85, 86, 88, 112]) were the second most targeted population, with some programs targeting those who had past or current involvement with child protective services (*n* = 5) [85, 99, 109, 119, 123]. One program targeted refugees (*n* = 1) [129]. Some programs’ target population was age specific (*n* = 8) [62, 75, 96, 98, 121], such as programming for teens (*n* = 4) [81, 98, 113, 115] and others focused on populations experiencing violence (*n* = 6), [78, 87, 100, 101, 113, 125], homelessness (*n* = 6) [78, 84, 96, 101, 123, 125], mental health and complex trauma (*n* = 5) [76, 84, 100, 113, 123], opioid addiction (*n* = 5) [71–74, 105]; and poverty and low-income (*n* = 4) [78, 81, 101, 113]. Only one program targeted fathers (*n* = 1) [118] in tandem with the PPWUD, with one program also supporting affected families (*n* = 1) [126].

The most reported purpose among programs was to prevent substance use during pregnancy (*n* = 33), closely followed by supporting healthy pregnancies (prenatal, perinatal, and postpartum), including parenting supports and positive child health outcomes (*n* = 31). The multifaceted needs of PPWUD were addressed by many of these programs through offering social services (e.g., housing, employment, child and family services) (*n* = 18) [63, 75, 78, 84, 89, 93, 97, 99, 104, 106, 108, 112, 116, 119, 130, 131] within the program or offering referrals, advocacy, and accompaniment to external services. A few programs specifically offered wrap-around services (*n* = 6) [65, 66, 71, 86, 90] including treatment services and personal support services, with two (*n* = 2) programs specifically supporting homeless pregnant people [82, 123]. Some programs did not report any purpose to their program intervention (*n* = 14) [76, 86, 90, 99, 124]. Other program purposes included harm-reduction (*n* = 11) [63, 64, 69, 78, 99, 101, 106, 116, 117, 126, 127], being culturally grounded and trauma-informed (*n* = 13) [68, 69, 76, 99–101, 105, 106, 126], treating mental health (*n* = 7) [69, 70, 84, 85, 100, 101, 108], supporting families in recovery (*n* = 5) [67–69, 112, 116], and supporting transition back to community (*n* = 5) [71, 78, 84, 126, 130].

### Program characteristics

The included articles provided a range of program characteristics, including mode of delivery, type of services offered, and who provided the services. A total of 61 articles (85.9%) provided information on program delivery. Programs were delivered in a variety of ways to meet the diverse needs of PPWUD and in some cases, programs were delivered in multiple modes. In-person programs (*n* = 28), such as those at community health centres or outpatient programs, and drop-in programs (*n* = 27) were most reported (for example see [73, 75, 77, 97–99, 102, 113, 129]). Several programs were delivered via outreach teams (*n* = 23) and in most cases these were street outreach teams (for example see [65, 81, 87, 89, 90, 93, 101, 109, 117, 119, 122, 123]). Additional programs included in-home visiting (*n* = 8) where service providers delivered a program in the home of the PPWUD [65, 101, 103, 104, 116, 125, 132, 133]. Some programs were delivered as 24/7 programs, including in-patient programs at a hospital or treatment centre (*n* = 10) or as residential and/or supportive housing programs (*n* = 13) [62, 64, 65, 68, 69, 71, 72, 74, 78, 79, 82–85, 105, 110, 116, 127, 130]. Only one program was noted as being delivered ‘virtually’ via phone [63].

The next most reported characteristic was program service providers (*n* = 58 articles, 81.7%). A total of 13 articles did not report information about service providers. In almost all articles, multiple providers were reported, and this was sometimes reflected by the reporting of “multidisciplinary teams” (*n* = 6) [67, 73, 84, 100, 105, 108]. Additionally, 21 articles (29.6%) reported that at least some staff members were people with lived/living experience of drug use (see Table 3 for details) (for example see [65, 69, 75, 77, 86, 92, 96, 101, 106, 121, 134]). Social service providers were most reported (*n* = 55) and included social workers, child welfare workers, and cultural supports (for example see [68, 90]). Program administration and support staff, such as office workers and group facilitators were next most mentioned (*n* = 52) (for example see [66, 67]). Medical and nursing staff were also frequently reported (*n* = 47 and *n* = 36 respectively). In addition to primary care physicians and registered nurses, PPWUD had access to a range of specially trained physicians and nurses, including, for example, paediatricians, addictions medicine physicians, obstetricians, maternal-foetal specialists, public health nurses and nurse practitioners (for example see [88, 94]). Psychiatrists were also frequently reported, and, in a few cases, psychiatrists specialised in reproductive psychiatry [88]. In addition to psychiatrists, other mental health professionals, such as counsellors, were reported (*n* = 27) (for example see [68, 102]). Interestingly, many PPWUD had access to allied health professionals (*n* = 33), including nutritionists (*n* = 8) [65, 66, 76, 88–90], dental professionals (*n* = 7) [65, 66, 76, 87, 90, 94] and speech pathologists (*n* = 6) [65, 89, 90]. Surprisingly, few birth-related specialists (*n* = 18), apart from obstetricians, were reported. Those that were reported included infant development specialists (*n* = 8) [65, 89, 90, 93, 94] and midwives and doulas (*n* = 6) [66, 68, 90, 101, 128, 133]. Most doulas were specifically for Indigenous PPWUD.

**Table 3:** Program service providers (reported by frequency)

Service Provider	Frequency (n)
**Social Services**	
Social worker	11^a^
Child welfare/CPS	10
Family worker	7^b^
Housing worker	4
Lawyer/legal advocate	4
Early childhood educator	4
Income assistance worker	4
Probation officer	2
Knowledge keeper	2
Volunteer baby cuddler	1
Cook	1
**Program Staff**	
Program staff	24
Patient care coordinator	11
Outreach worker	9
Mentor	7
**Physicians**	
Primary care physicians	20
Obstetrician	8
Paediatrician	6
Psychiatrist	6^c^
Addiction medicine	4
Maternal-foetal specialist	2
**Nursing**	
Public health nurse	18
Registered nurse	12
Nurse practitioner	6
**Mental health**	
Therapist	15
Trauma counsellor	3
Child psychologist	1
**Harm reduction/treatment**	
Addiction counsellor	9
**Allied Health**	
Nutritionist	8
Cultural support	7
Dental hygienist	7
Speech pathologist	6
Recreation therapist	2
Art therapist	2
Occupational therapist	2
Physiotherapist	2
Music therapist	1
Pharmacist	1
Registered massage therapist	1
Acupuncturist	1
**Birth-related supports**	
Infant Development Specialist	8
Lactation consultant	3
Doula	3^d^
Midwives	3

Types of services providers in programs for pregnant people who use drugs in Canada (by frequency)

^a^Includes 1 NICU social worker

^b^Includes 2 Indigenous family workers

^c^Includes 2 reproductive psychiatrists

^d^Includes 2 Indigenous doulas

Finally, all articles reported on the types of services provided. We have provided the most frequently reported services in this text and the frequency of all services can be found in Table 4. Articles most frequently mentioned programs that offered ‘wrap-around’ services (*n* = 58) (for example see [62, 90, 105]). Wrap-around services involve multiple services being offered in one location. Social services were the most frequently mentioned service (*n* = 71) and entailed a range of supports for PPWUD, including the provision of food and basic needs (*n* = 39) (for example see [83, 85, 90, 123]), child welfare or child custody services (*n* = 38) (for example see [86, 112, 122, 132]), housing services (*n* = 28) (for example see [70, 81, 82, 104, 112, 134]), transportation or public transit passes (*n* = 21) (for example see [76, 101, 116, 119, 126]), and cultural practices including connections to Elders or access to traditional ceremonies (*n* = 20) (for example see [66, 68, 86, 90, 133]).

**Table 4:** Services provided by programs (reported by frequency)

Provided Service	Frequency (n)
**Social Services**	
Food and basic needs	39
Child welfare/custody services	33
Housing	28
Transportation/transit passes	21
Cultural practices/ceremonies	20
General life support/social work	17
Referrals to other community organisations/services	16
Child minding	13
Legal education/advocacy	11
Income assistance/financial navigation	9
Peer connections	9
Employment/training/education	5
Clothing	5
Rent supplements	3
Spiritual care	2
Social events	1
**Harm reduction/treatment**	
Counselling	37
Healthcare including withdrawal management	18
Individual recovery planning/education	17
Groups	14
Referral to other programs	9
Opioid Agonist Treatment	5
Psychosocial support	5
Not specified	3
Peer support	1
Cultural support	1
**Mental health**	
General services/not specified	24
Counselling	22
Groups	8
Psychiatry	7
Music/art/pet therapy	4
Referral to residential treatment	4
Referral to community groups	3
Mental health nursing support	3
Yoga	1
Peer support	1
**Prenatal Services**	
Healthcare services	35
Classes	13
General support/not specified	10
Groups/support groups	8
Referrals to other services	7
Material support (e.g. vitamins, vouchers)	6
Family and birth planning	3
Home visits	3
Psychological support	2
Cultural practices	1
**Postnatal Services**	
Healthcare services	22
Groups	15
Classes	13
Support/discharge planning	10
Child healthcare	7
Childcare	6
Home visits	3
Transportation to postpartum appointments	3
Baby cuddling	2
Equipment (e.g. car seat, diapers)	2
Housing coordination	1
Respite for parent	1
Psychological support	1
Cultural practices	1
**Reproductive and sexual health services**	
Contraception	10
Healthcare services, including infertility care	7
STBBI^a^ testing and care	6
Pregnancy planning and abortion	2
Groups	1
Pregnancy testing	1
Reproductive psychiatry	1
Not specified	1
**Physical health**	
Primary care	16
Children’s health	13
General healthcare services	13
Public health services	8

Breakdown of services provided in programs for pregnant people who use drugs in Canada (by frequency)

^a^Sexually transmitted and blood borne infections

Harm reduction and treatment services were the next most frequently mentioned service (*n* = 64). Many programs offered substance use-specific counselling (*n* = 37) (for example see [63, 87, 88, 90, 100, 101, 117, 124]). Some offered healthcare including access to in-patient treatment programs (*n* = 18) (for example see [62, 115, 117]) and few also offered opioid agonist treatment (OAT) onsite (*n* = 5) (for example see [71, 80]). Other common forms of harm reduction and treatment support included individual recovery planning (*n* = 17) (for example see [66, 131]), group education (*n* = 14) (for example see [76, 79, 101]), and general harm reduction support (*n* = 12) (for example see [67, 81, 118]).

Not surprisingly, prenatal, perinatal, and postnatal services were frequently mentioned in the literature. We defined prenatal services as services that were specifically offered pre-childbirth and perinatal services as services that were offered during pregnancy and post-birth. Prenatal services (*n* = 56) most frequently included health care including from primary care physicians and obstetricians (*n* = 35) (for example see [76, 126]), as well as education through classes (*n* = 13) (for example see [78, 119, 123]) and support groups (*n* = 8) (for example see [66, 90]). Although not as commonly mentioned, perinatal services (*n* = 20) were highlighted in the literature and most involved the provision of healthcare services (*n* = 10) (for example see [124, 127, 130]). Postnatal services were also frequently mentioned (*n* = 51) and mostly included postpartum healthcare (*n* = 22) (for example see [65, 86, 89, 98]) and opportunities for education and support through groups (*n* = 15) and classes (*n* = 13) (for example see [88, 90, 115]). Perhaps more surprisingly, SRH services were rarely explicitly mentioned (*n* = 19). Articles that did mention provision of SRH services often referred to contraception (*n* = 10) (for example see [68, 92, 94, 126]) and healthcare services (*n* = 7) (for example see [90, 93, 127]).

Mental and physical health care were referenced throughout the included articles. Mental healthcare was most frequently reported (*n* = 54), although most articles did not specify the type of mental health support being provided (*n* = 24). The most common specified form of support was counselling (*n* = 22) (for example see [63, 90, 119, 124]). Physical healthcare was mentioned less frequently (*n* = 23), although some healthcare services may have been reported under other types of services (e.g., prenatal, postnatal). Primary care (*n* = 16) (for example see [75, 90, 98]), children’s healthcare (*n* = 13) (for example see [66, 121]), and general healthcare services (*n* = 13) (for example see [67, 88, 98]) were most provided by the identified programs.

### Program outcomes and employed practices

Out of the 71 articles reviewed, 16 did not specifically report outcomes. The remaining 55 articles reported more than one program outcome as measured through program evaluations. The most frequently reported program outcome was keeping birthing parent and baby together (*n* = 18) (for example see [66, 88]). Additional positive outcomes included better connections with services (*n* = 14) (for example see [68, 108]), reducing substance-related harms for birthing parent and baby (including abstinence from substances) (*n* = 14) (for example see [88, 128]), reducing service fragmentation (*n* = 12) (for example see [65, 72]), empowering PPWUDs and/or new parents (*n* = 8) (for example see [71, 74, 114]); and a reduction in judgement and stigma from service providers towards PPWUDs (*n* = 5) (for example see [73, 94]). Using substances during pregnancy increases the risk of maternal and pre- and postnatal health issues, therefore many of the programs identified within the literature had a specific focus on maternal and prenatal health, with a drive to reduce negative birth outcomes for birthing-parent and baby. Specific health-related outcomes were medical needs attended to (*n* = 11) (for example see [100, 127]), a reduction in negative birth outcomes and an increase in positive health outcomes for baby (*n* = 7) (for example see [95, 98]), attending to the general needs of the birthing-parent and baby (*n* = 5) (for example see [86, 87, 119]) and meeting basic needs of PPWUD (*n* = 4) (for example see [90, 98]). It can be argued that the driving force behind the implementation of programs that are tailored to support PPWUD is long-term positive change for families. To achieve continuous abstinence, healing and sound parenting, new practices and skills need to be adopted by birthing parents. Other outcomes therefore included the development of new skills and healthier behaviours (*n* = 7) (for example see [67, 88]), improved support for Indigenous PPWUDs (*n* = 3) [66, 86], strengthened parent knowledge and skills (*n* = 5) (for example see [75, 97]), increase in contraception use (*n* = 1) [126], increase in positive relationships with service providers (*n* = 3) [98, 133], reduction in arrests (*n* = 1) [126] and seeking or attending higher education (*n* = 1) [126].

All articles that reported practices employed in the programs (*n* = 54) noted utilising more than one method to support PPWUD. The most common practices used by service providers included non-judgmental care (*n* = 13) (for example see [62, 98, 133]), community-based support (*n* = 12) (for example see [117, 125]), harm-reduction strategies (*n* = 12) (for example see [108, 123, 133]) and trauma-informed care (*n* = 11) (for example see [86, 114]). Many of the practices adopted work to reduce barriers to services for PPWUD and include collaboration between service providers (*n* = 8) (for example see [76, 117]), accessible childcare (*n* = 6) (for example see [75, 134]) and home visits (*n* = 3) [119, 131]. Culturally informed care (*n* = 9) (for example see [63, 98]) was noted, with several programs working with Indigenous elders and community members to integrate cultural practices, such as smudging, ceremonies and prayer into wellbeing and rehabilitation activities. Embedded in some of the programs were positive approaches, which included goal-oriented strategies (*n* = 7) (for example see [66, 135]), and strengths-based practices (*n* = 5) (for example see [68, 128]) that clients could focus on to be self-reliant and to help themselves. Gender was a common theme identified, with six articles noting women-centred care as being central to the program discussed, with no programs focusing on other diverse gender identities. The least recorded practices were mentorship (*n* = 2) [114, 128], parent-baby togetherness (*n* = 2) [66, 68], family-oriented care (*n* = 2) [100, 133], meeting the client where they are at (*n* = 1) [108], non-pharmacological interventions (i.e. swaddling, quiet environments) (*n* = 1) [73] and absence of ‘drug talk’ (*n* = 1) [68]. Although these practices were discussed only a few times, it is possible that they were also employed in other programs but not mentioned. In total, 17 articles did not report any specific practices employed within the program.

The diversity and breadth of practices employed by programs and service providers showcase the multitude of knowledge, services and resources required for rehabilitation, maternal and pre/post-natal care, and trauma-healing. However, the programs are not without challenges. Out of the 71 articles, 21 specifically reported challenges identified within the programs. Specific to service providers and resources, issues related to tensions between service providers (*n* = 4) (for example see [90, 133]), staff and service provider shortages (*n* = 3) [65, 96], limited group sessions (*n* = 1) [131], limited one-on-one sessions (*n* = 1) [131], program length restrictions (*n* = 1) [131], limited partnerships with service providers (*n* = 1) [131], limited physical space (*n* = 1) [131], limited options for home visitations (*n* = 1) [119], limited funding (*n* = 1) [65] and limited accessible locations (*n* = 2) [131, 132] were reported. Overarching these challenges is funding and without sufficient capital, programs that support vulnerable groups, particularly operated by non-profit organisations, are limited in the support and resources they can offer. Another key challenge identified in the review were issues related to program access. Barriers to access for PPWUD included overcoming stigma and shame (*n* = 6) (for example see [71, 72, 106]), fear of their child(ren) being apprehended and entered into care of the state (*n* = 4) (for example see [72, 88]), a lack of trust in the system (*n* = 3) (for example see [97, 126]) and limited ‘safe’ service providers (*n* = 1) [77]. These challenges could also be grouped with the long-term effects of colonialism which were twice reported as being challenges [97, 99]. Historical and ongoing prejudice and discrimination towards Indigenous peoples has impaired trust of agencies and services. Lastly, it was reported that there is a lack of tailored support for men (*n* = 1) [128] and youth (*n* = 1) [128], variability in addressing other barriers to care (i.e. transportation, childcare) (*n* = 3) [131, 132], a need to tackle misinformation about pregnancy (*n* = 1) [106] and nation-wide variations in how data are collected (*n* = 1) [128].

## Discussion

The objective of this scoping review was to present information about existing programs that support PPWUD’s access to SRH services in Canada. An examination of the programs’ context, characteristics and outcomes provided insight into the services available across Canada, their accessibility, purpose, and practices. Our findings highlight the existence of some programs and practices for PPWUD that focus on integrated care, harm reduction, wrap-around services, trauma-informed care, culturally-grounded services, and peer-to-peer models. These diverse program offerings speak to the unique medical and social implications of drug use among pregnant people, including maternal health, foetal development, withdrawal symptoms, parental rights with risks of child apprehension through social services, long-term family impact and crucially, stigmatisation from society, health care providers and at times, PPWUD’s own social supports—creating feelings of isolation and barriers to seeking prenatal care and substance use supports.

Encouragingly, 46 unique programs for PPWUD were identified in our review; however, few peer-reviewed articles were available, underscoring how this vulnerable population is underrepresented in research. Notably, many programs provided a range of health and social services that support SRH but did not explicitly refer to themselves as SRH services (e.g. primary care for PPWUD). Still, we reported on these programs as they inform the sexual and reproductive health and wellbeing of parent and baby. The range of programs delivered outside of SRH-speciality care underscores the need for cross-sector collaboration to support PPWUD. SRH care for this population cannot be adequately addressed solely through maternal and infant health care, and this review highlights examples of how other health and social service providers have collaborated for more holistic care, particularly through wrap-around services. Additionally, the length of these programs presents crucial questions about long-term support for this population. With many of these programs funded provincially, the duration of programs and their sustainability rely on provincial leaders and are left to the political will of the parties in power.

Most programs and services for PPWUD were concentrated in urban areas and in Western Canada. Large disparities and limited evidence in program availability were found in the Atlantic provinces (New Brunswick, Newfoundland and Labrador, Nova Scotia, Prince Edward Island) and the territories (The Northwest Territories, Yukon, and Nunavut) where fewer to no services were identified. Most of the programs and services were in the province of British Columbia, with Vancouver reporting the most programs in the country. Vancouver has been the epicentre of the toxic drug crisis, with multiple factors contributing to this crisis including poverty, racism, trauma, colonial violence, and geographic location to drug trade routes [136]. In 2003, *Insite,* the first supervised consumption site for people who inject drugs in North America was opened in Vancouver’s Downtown Eastside. The successful policy implementation through multi-level governance to address the growing opioid crisis using a harm reduction approach placed Vancouver at the forefront of the toxic drug crisis in North America [136–138]. This shift in conceptualising drug use as a criminal issue to a health issue gave precedence for more harm reduction programs and safe-injection sites to open across Canada [136, 139]. Despite these efforts, an increase of fentanyl and other deadly contaminants in the drug supply contributed to British Columbia declaring a public health emergency in 2016 [140]. The province continues to experience the highest number of drug-related deaths in Canada [3].

In the Atlantic provinces, there have been widespread concerns about limited access and availability of comprehensive SRH services for all people of reproductive age. These challenges affect various aspects of reproductive health, including access to contraception, prenatal care, abortion services, fertility treatments, and specialised care for certain reproductive health conditions [141]. Adding to these challenges, the reproductive justice landscape in the Atlantic region is closely bound to conservative Catholicism and the rise of conservative politics in the region, all reflected in the underfunding of SRH services [142]. In 2021, New Brunswick was found in violation of the Canada Health Act by refusing to fund abortion clinics, creating distressing implications considering the province’s rural landscape and lack of public service points [141]. New Brunswick’s refusal to fund abortion clinics is outside of the scope of this review, but it is a notable example of the ways that vital SRH services are underfunded, continuing to create barriers for marginalised communities in the Atlantic region.

The Northern territories present similar access barriers to vital services, with no programs identified in this review. However, we note that this does not negate that supportive services exist in the Northern territories. Some services focusing on foetal alcohol syndrome and domestic violence prevention were identified as outside of the scope of this review [143]. This is concerning given that high drug mortality rates have been identified in the region, prompting the Yukon to declare a state of emergency in 2022 [144]. The Northern territories represent unique intersections that present challenges for service delivery including location (I.e. remote and rural geography), funding (e.g. healthcare worker shortage), and large Indigenous populations that experience compound systematic barriers with a lack of basic social services including housing, food and water insecurity, and healthcare [145]. Many of the programs and services funded in Canada for PPWUD serve Indigenous communities with culturally-grounded philosophies and mandates of self-determination, sovereignty, and healthy communities.^2^ While grassroots organisations are delivering essential healthcare to marginalised populations, it is also the responsibility of territorial, provincial and federal governments to ensure appropriate and sufficient care is provided to this population. Further exploration into services and programs for PPWUD in the territories could provide understanding into the barriers and needs, as well as the historical, social, and cultural realities that shape this population's lived experience. Investing in programs would ensure more PPWUD could access essential services and could lead to reduced family separation and drug-related deaths.

Canada still has much to learn in terms of equitable program design and delivery and could gain valuable knowledge from programs that provide national guidelines for pregnancy care. In Australia, there is a national standard of care to ensure that pregnant people are provided with “consistent, high-quality, evidence-based, maternity care” including specific guidelines for substance use in pregnancy [38]. The guidelines for substance use in pregnancy provide five strategic and clinical approaches to assessing substance use in pregnant people, with an emphasis on a holistic approach that centres the pregnant person’s well-being, safety, and family [38]. The implementation of similar national guidelines in Canada could reduce geographical inequities and support standardised care across the country. Similarly, grassroots initiatives in international communities with high rates of HIV/AIDS may provide a learning opportunity for Canada on how to provide and integrate SRH to priority populations in resource-constrained settings amidst an urgent public health crisis [146]. Through these programs an emphasis is put on creating non-judgemental space for sensitive discussions, peer-led community empowerment, including diverse and marginalised groups (2SLGBTQIA + , sex workers), and ensuring that all services are accessible for family members and children [146].

SRH services for pregnant Indigenous populations across Canada are limited. In response to this inequity, the Society of Obstetricians and Gynaecologists put forth a policy report to highlight the realities of access to SRH services for Indigenous people in Canada [147]. Maternal services for this population are described as being in a “state of emergency”, with a lack of culturally safe services that address and understand compound stigma, harms of environmental toxins on maternal and child health, high rate of sexually transmitted diseases, high-risk pregnancies, maternal mortality, substance use disorder, community disintegration and political marginalisation [147]. These complex social determinants of health are the result of historical and ongoing colonisation that contributes to poor health outcomes for pregnant Indigenous populations [147]. Culturally safe care for this population is crucial, as mistrust of health services and medicalization is a barrier to this community that continues to face systemic violence through historical and ongoing practices of forced sterilisation, and threats of child apprehension from child welfare services [147]. Many programs for PPWUD in Canada target Indigenous populations and (re)connecting to cultural roots and practices is considered to have profound positive outcomes for Indigenous people. Many draw strength from these connections which support healing [148]. However, these programs continue to present accessibility barriers with most programs concentrated in urban areas, leaving gaps in culturally appropriate services that are available to Indigenous PPWUD living outside of urban centres. Future research with Indigenous PPWUD must incorporate a reproductive justice framework to ensure reproductive self-determination, bodily autonomy, the right to choose to parent (or not), and culturally specific practices for health, community, education, and family are prioritised [7, 40–43, 46].

The absence of programs that target 2SLGBTQIA + populations also present a gap in knowledge. 2SLGBTQIA + populations experience higher levels of substance use, translating into compound stigma. Compound stigma is the ‘cumulative impact’ of being a member of one, or many, marginalised groups (e.g., racial/ethnic minority, 2SLGBTQIA +), and experiencing addiction, mental illness, and complex trauma, each transmitting its own social stigma [148, 149]. Many 2SLGBTQIA + people are marginalised and excluded from mainstream healthcare practices, including SRH services and programs for PPWUD. These exclusions, we argue, will delay Canada from meeting SDGs 3, 5 and 11. Programs and services could be strengthened by addressing the unique needs of this population, the role of compound stigma, and how stigma affects service access and interaction [149].

The geographic disparities strongly suggest that Canada is likely to fall short of meeting SDGs 3, 5 and 11. What is apparent in this review is the importance of multidisciplinary care for PPWUD and ensuring their needs are met for them and their children to live healthy lives. It can be argued that *access* to essential services and resources is *the* fundamental element to population health promotion. Although the importance of access to services (such as healthcare, reproductive health, and housing) is highlighted in the UN SDGs [48] it is not included in the FSDS [50]. As many of the programs that support PPWUD are funded by provincial governments or through non-profit organisations, it suggests a lack of commitment from the Federal Government on meeting SDGs 3, 5 and 11 and provides an unsustainable foundation for many of the programs to operate. Currently, many of the programs are relying on the political will of provincial governments (which vary by province) and the extensive efforts of securing private and/or public funding. Overall, recognizing and responding to the unique situations and needs of marginalised and vulnerable populations is essential for Canada.

### Relevance and importance to the field

PPWUD are often overlooked in the conversation on improving parent and children’s health, despite being at high risk for birth complications and other health harms. Nevertheless, we identified many program strengths, including the practice of connecting with other organisations and service providers in the community. The power of partnership and connection was apparent in many of the services and points to the importance of programs being well connected to maximise their reach. The practices and program components identified in this review may inform adaptations in other jurisdictions.

Perhaps not surprisingly, we also identified several areas where services must be improved to make services equitable for all PPWUD. There were geographical disparities in availability of programs both within and between provinces and territories. At a practical level, there is an urgent need to expand services for PPWUD in rural, remote, and Northern communities, as well as underserved provinces and territories. This expansion is imperative as Indigenous peoples continue to be disproportionately affected by the toxic drug supply [150] and many reside in remote communities and form a large proportion of the population in the territories [151].

Secondly, the initial aim of this review was to identify programs and practices that support access to SRH services. Yet, we noted that many of the included programs did not explicitly mention SRH services apart from pre- and postnatal care. Those that did mention specific services tended to focus on contraception and family planning. There was also only one program that referred to pregnancy options. The lack of SRH services to support PPWUD was disappointing and points to ongoing reproductive justice challenges for this population, particularly in terms of autonomy over reproductive decisions. Finally, most of the services only served PPWUD and very few provided services to their partner or families. It is important to include family members in services to some degree as they are often key support for PPWUD after they leave a service provider. Moving forward, service providers should consider expanding the range of SRH services available to PPWUD and consider how to meaningfully include family members.

### Limitations

Given that scoping reviews aim to characterise the availability and landscape of literature on a specific topic, this review is limited in analysis or evaluation of programs available to PPWUD across Canada. For instance, major events, including COVID-19 and natural disasters such as wildfires, may impact the ideal delivery of services; however, this analysis was beyond the scope of this review [152]. While every effort was made to find all relevant literature on available programs, there is the possibility that information on some programs is not publicly available or there is limited available evidence and therefore these programs are not included in the review. The search also focused on programs that explicitly focused on unregulated drug use. Therefore, programs that focused only on alcohol, cannabis, or tobacco were not included. It is possible that some of these programs may support PPWUD even though it is not part of their directive. Further, we found that some of the articles, particularly the grey literature, lacked program details and therefore, there is some variation between the comprehensiveness of the reporting across the programs. As an example, some articles detailed each service provider who works with a specific program while some articles did not report any. Moreover, as the focus was literature published from 2016–2023, it is unknown if there were any programs that supported PPWUD prior to 2016 that have ceased operating or any potential operation impacts stemming from COVID-19. Finally, many of the articles discussed the same programs, particularly the more well-known programs.

## Conclusions

Access to tailored programs that provide holistic support for PPWUD’s health and social needs are a lifeline for this structurally vulnerable population. Across Canada, there are a diversity of programs ranging from in-patient programs to comprehensive wrap-around community-based programs. Identified in the scoping review, which echoes previous research on wrap-around services for PPWUD [65], is the importance of single-entry service where PPWUD can access the resources they require ‘under one roof’. There is a need for judgement-free care for PPWUD and holistic approaches to recovery and parenting. Although many of the programs detailed in the scoping review reported positive outcomes for PPWUD, there remains opportunities to improve programs, such as expanding geographic availability and range of services offered. Furthermore, many of the programs are in response to the need for immediate care for PPWUD, thus, there is a lack of, and necessity for more preventative strategies. Adhering to meeting the needs of PPWUD and ensuring healthy pregnancies and births across Canada will contribute to attaining healthy lives for all ages (SDG 3), gender equality (SDG 5) and inclusive, safe, and resilient communities (SDG 11). Future research should consider client perspectives and experiences of these programs and the impacts on communities with minimal access to such supports.

### Supplementary Information

**Additional file 1. **Sustainable Development Goals relevant to pregnant people who use drugs.

**Additional file 2. **Search strategy.

## Data Availability

All data generated or analysed during this study are included in this published article.

## Abbreviations

PPWUD: Pregnant people who use drugs
SDG: Sustainable Development Goals
SRH: Sexual and reproductive health
HIV: Human Immunodeficiency Virus
SRHR: Sexual and reproductive health and rights
FNIM: First Nations, Métis, and Inuit communities
FSDS: Federal Sustainable Development Strategy
2SLGBTQIA +: Two-Spirit, Lesbian, Gay, Bisexual, Transgender, Queer or Questioning, Intersex, Asexual and additional sexual orientations and gender identities

## Footnotes

1 Much of the existing literature on substance use and pregnancy conceptualises gender as a binary with only cisgender women being able to become pregnant; thus, our summary of existing evidence refers primarily to cisgender women. However, we recognize that gender exists across a spectrum and pregnancy can be and is experienced by people with diverse gender identities.

2 See The Cedar Project (https://cihr-irsc.gc.ca/e/52200.html), Pauktuutit Inuit Women of Canada (https://pauktuutit.ca/), The Native Women’s Association of Canada (https://nwac.ca/), Les Femmes Michif Otipemisiwak (https://metiswomen.org/), Mothering Project (https://www.mountcarmel.ca/how-we-help/early-learning-parenting/), Sheway (https://www.vch.ca/en/service/sheway-pregnancy-outreach-program), H.E.R (https://www.catie.ca/programming-connection/the-her-pregnancy-program).

## References

[1] United Nations Office on Drugs and Crime, editors. World drug report, 2022. New York: United Nations; 2022. Available from https://www.unodc.org/res/wdr2022/MS/WDR22_Booklet_2.pdf. [Cited 2023 July 19].

[2] Mburu G, Ayon S, Mahinda S, Kaveh K (2020). Determinants of women's drug use during pregnancy: Perspectives from a qualitative study. Matern Child Health J.

[3] Public Health Agency of Canada. Opioid and stimulant related harms in Canada. 2023. Available from: https://health-infobase.canada.ca/substance-related-harms/opioids-stimulants/. [Cited 2023 July 22].

[4] Paradis D. Treaty 6 Chiefs declare state of emergency over opioid deaths. APTN. 2023. Available from: https://www.aptnnews.ca/national-news/treaty-6-chiefs-declare-state-of-emergency/. [Cited 2023 July 29].

[5] Government of British Columbia. Provincial health officer declares public health emergency. Government of British Columbia. 2016. Available from https://news.gov.bc.ca/releases/2016HLTH0026-000568. [Cited 2023 July 30].

[6] Kar P, Tomfohr-Madsen L, Giesbrecht G, Bagshawe M, Lebel C (2021). Alcohol and substance use in pregnancy during the COVID-19 pandemic. Drug Alcohol Depend.

[7] Muckle G, Laflamme D, Gagnon J, Boucher O, Jacobson JL, Jacobson SW (2011). Alcohol, smoking, and drug use among Inuit women of childbearing age during pregnancy and the risk to children. Alcohol Clin Exp Res.

[8] Niccols A, Dell CA, Clarke S (2010). Treatment issues for Aboriginal mothers with substance use problems and their children. Int J Ment Health Addict.

[9] Kolahdooz F, Nader F, Yi KJ, Sharma S (2015). Understanding the social determinants of health among Indigenous Canadians: priorities for health promotion policies and actions. Glob Health Action.

[10] Falk J, Dahl M, Raymond CB, Chateau D, Katz A, Leong C, Bugden S (2017). Opioid use during pregnancy: a population-based cohort study. CMAJ Open.

[11] Levander XA, Foot CA, Magnusson SL, Cook RR, Ezell JM, Feinberg J (2023). Contraception and healthcare utilization by reproductive-age women who use drugs in rural communities: a cross-sectional survey. J Gen Intern Med.

[12] Logue TC, Wen T, Friedman AM (2022). Demographic trends associated with substance use disorder and risk for adverse obstetric outcomes with cannabis and opioid use disorders. J Matern Fetal Neonatal Med.

[13] MacAfee LK, Harfmann RF, Cannon LM, Minadeo L, Kolenic G, Kusunoki Y (2020). Substance use treatment patient and provider perspectives on accessing sexual and reproductive health services: Barriers, facilitators, and the need for integration of care. Subst Use Misuse.

[14] Meschke LL, McNeely C, Brown KC, Prather JM (2018). reproductive health knowledge, attitudes, and behaviors among women enrolled in medication-assisted treatment for opioid use disorder. J Womens Health (Larchmt).

[15] Owens L, Micks E, Moreno C, Glick S (2020). Reproductive health care utilization by women who inject drugs and exchange sex in the Seattle area. Subst Use Misuse.

[16] Duff P, Shoveller J, Zhang R, Alexson D, Montaner JS, Shannon K (2011). High lifetime pregnancy and low contraceptive usage among sex workers who use drugs- an unmet reproductive health need. BMC Pregnancy Childbirth.

[17] Iversen J, Page K, Madden A, Maher L (2015). HIV, HCV, and health-related harms among women who inject drugs: Implications for prevention and treatment. J Acquir Immune Defic Syndr.

[18] Mburu G, Ndimbii J, Ayon S, Mlewa O, Mbizvo M, Kihara C (2018). Contraceptive use among women who inject drugs: motivators, barriers, and unmet needs. Womens Reprod Health.

[19] Weber AE, Tyndall MW, Spittal PM, Li K, Coulter S, O'Shaughnessy MV, Schechter MT. High pregnancy rates and reproductive health indicators among female injection-drug users in Vancouver, Canada. Eur J Contracept Reprod Health Care. 2003;8(1);52–8. Available at: https://pubmed.ncbi.nlm.nih.gov/12725675/. [Cited 2023 Apr 14]. 12725675

[20] Sales P, Murphy S (2000). Surviving violence: Pregnancy and drug use. J Drug Issues.

[21] Heaman MI, Martens PJ, Brownell MD, Chartier MJ, Thiessen KR, Derksen SA (2018). Inequities in utilization of prenatal care: a population-based study in the Canadian province of Manitoba. BMC Pregnancy Childbirth.

[22] Martin CE, Parlier-Ahmad AB, Beck L, Jain V, Terplan M (2022). A comparison of sex-specific reproductive and sexual health needs between addiction medicine and primary care treatment settings. Subst Use Misuse.

[23] Nussey L, Hunter A, Krueger S, Malhi R, Giglia L, Seigel S (2020). Sociodemographic characteristics and clinical outcomes of people receiving inadequate prenatal care: A Retrospective cohort study. J Obstet Gynaecol Can.

[24] Shalev C. Rights to Sexual and Reproductive Health. United Nations. 1998. Available from www.un.org/womenwatch/daw/csw/shalev.htm. [Cited 2023 July 22].

[25] Bornstein M, Berger A, Gipson JD (2020). A mixed methods study exploring methadone treatment disclosure and perceptions of reproductive health care among women ages 18–44 years, Los Angeles, CA. J Subst Abuse Trea.

[26] Foti TR, Cragun D, Mackie J, Agu N, Bell M, Marshall J (2023). Personas of pregnant and parenting women with substance use and their barriers and pathways to system engagement. Birth.

[27] Gibson B, Hoff E, Haas A, Adams ZM, Price CR, Goddard-Eckrich D, Sheth SS, Dasgupta A, Meyer JP (2022). Overlapping needs for sexual and reproductive health and HIV prevention in women with substance use disorders. Womens Health (Lond).

[28] Knight KR (2020). Structural factors that affect life contexts of pregnant people with opioid use disorders: The role of structural racism and the need for structural competency. Women Reprod Health.

[29] Wright TE (2019). Integrating reproductive health services into opioid treatment facilities: A missed opportunity to prevent opioid-exposed pregnancies and improve the health of women who use drugs. J Addict Med.

[30] Owens L, Gilmore K, Terplan M, Prager S, Micks E (2020). Providing reproductive health services for women who inject drugs: a pilot program. Harm Reduct J.

[31] Ayon S, Jeneby F, Hamid F, Badhrus A, Abdulrahman T, Mburu G (2019). Developing integrated community-based HIV prevention, harm reduction, and sexual and reproductive health services for women who inject drugs. Reprod Health.

[32] Ayon S, Ndimbii J, Jeneby F, Abdulrahman T, Mlewa O, Wang B (2018). Barriers and facilitators of access to HIV, harm reduction and sexual and reproductive health services by women who inject drugs: role of community-based outreach and drop-in centers. AIDS Care.

[33] Klaman SL, Lorvick J, Jones HE (2019). Provision of and barriers to integrating reproductive and sexual health services for reproductive-age women in opioid treatment programs. J Addict Med.

[34] Laks J, Walley AY, Bagley SM, Barber CM, Gaeta JM, Neville LA (2023). Developing a Women's Health track within addiction medicine fellowship: reflections and inspirations. Addict Sci Clin Pract.

[35] Sorsa M, Hohenthal M, Pikulinsky M, Sellergren H, Puura K (2023). Qualitative description of outreach and engagement in perinatal substance treatment in Finland. Subst Abuse Treat Prev Policy.

[36] Thompson TA, Ahrens KA, Coplon L (2020). Virtually possible: using telehealth to bring reproductive health care to women with opioid use disorder in rural Maine. Mhealth.

[37] Torchalla I, Linden IA, Strehlau V, Neilson EK, Krausz M (2015). "Like a lots happened with my whole childhood": violence, trauma, and addiction in pregnant and postpartum women from Vancouver's Downtown Eastside. Harm Reduct J.

[38] Government of Australia. Pregnancy care guidelines: Substance use. Canberra: Department of Health and Aged Care; 2019. Available from https://www.health.gov.au/resources/pregnancy-care-guidelines/part-c-lifestyle-considerations/substance-use. [Cited 2023 November 16].

[39] Government of Western Australia. Women and newborn drug and alcohol service (WANDAS). Perth: Healthy Western Australia; 2023. Available from https://www.healthywa.wa.gov.au/Articles/U_Z/Women-and-Newborn-Drug-and-Alcohol-Service-WANDAS. [Cited 2023 November 16].

[40] Idriss-Wheeler D, El-Mowafi IM, Coen-Sanchez K, Yalahow A, Yaya S (2021). Looking through the lens of reproductive justice: the need for a paradigm shift in sexual and reproductive health and rights research in Canada. Reprod Health.

[41] MacKenzie, Holly A. Reproductive Politics: Reproductive Choice to Reproductive. In: Justice Morrow MH, Hankivsky O, Varcoe C. Women’s health in Canada : challenges of intersectionality. Second edition. Morrow MH, Hankivsky O, Varcoe C, editors. Toronto; University of Toronto Press; 2022. p.148–161.

[42] Shahram SZ, Bottorff JL, Oelke ND, Dahlgren L, Thomas V, Spittal PM (2017). The cedar project: Using Indigenous-specific determinants of health to predict substance use among young pregnant-involved Indigenous women in Canada. BMC women’s health.

[43] Ross LJ (2017). Reproductive justice as intersectional feminist activism. Souls (Boulder, Colo).

[44] Canada FASD Research Network’s Action Team on Prevention from A Women’s Health Determinants Perspective. Supporting pregnant women who use substances. Vancouver: CanFASD; 2013. Available from https://canfasd.ca/wp-content/uploads/2016/09/What_Communities_Are_Doing_to_Help_February_7_2013.pdf. [Cited 2023 July 26].

[45] Walter M, Anderson C (2016). Indigenous statistics: a Quantitative research methodology.

[46] Allen L, Wodtke L, Hayward A, Read C, Cyr M, Cidro J. Pregnant and early parenting Indigenous women who use substances in Canada: A scoping review of health and social issues, supports, and strategies. J Ethn Subst Abuse. 2022:1–31 10.1080/15332640.2022.2043799. Epub ahead of print. PMID: 35238726. 10.1080/15332640.2022.2043799 35238726

[47] Proulx KR, Ruckert A, Labonté R (2017). Canada’s flagship development priority: maternal, newborn and child health (MNCH) and the Sustainable Development Goals (SDGs). Can J Dev Stud.

[48] United Nations Department of Economics and Social Development. The 17 Goals. New York: United Nations; 2023. Available from: https://sdgs.un.org/goals. [Cited 2023 July 23].

[49] McArthur JW, Rasmussen K (2019). Classifying Sustainable Development Goal trajectories: A country-level methodology for identifying which issues and people are getting left behind. World Dev.

[50] Government of Canada. The federal sustainable development strategy. Ottawa (ON): Government of Canada; 2020. Available from https://www.fsds-sfdd.ca/en. [Cited 2023 July 23].

[51] The Sustainable Development Report. Implementing the SDG stimulus. Dublin: Sustainable Development Report; 2023. Available from: https://dashboards.sdgindex.org/profiles. [Cited 2023 November 16].

[52] United Kingdom of Great Britain and Northern Ireland. Voluntary National Review of progress towards the Sustainable Development Goals. London: United Kingdom; 2019. Available from: https://assets.publishing.service.gov.uk/media/5d28c296ed915d2feeac499e/UK-Voluntary-National-Review-2019.pdf. [Cited 2023 November 16].

[53] United Nations Department of Economic and Social Affairs. Achieve gender equality and empower all women and girls. New York: United Nations; 2019. Available from https://sdgs.un.org/goals/goal5. [Cited 2023 November 16].

[54] Wolfson L, Schmidt RA, Stinson J, Poole N (2021). Examining barriers to harm reduction and child welfare services for pregnant women and mothers who use substances using a stigma action framework. Health Soc Care Community.

[55] Khachikian T, Amaro H, Guerrero E, Kong Y, Marsh JC (2022). Disparities in opioid treatment access and retention among women based on pregnancy status from 2006 to 2017. Drug Alcohol Depend Rep.

[56] Tran EL, England LJ, Park Y, Denny CH, Kim SY (2023). Systematic review: Polysubstance prevalence estimates reported during pregnancy, US, 2009–2020. Matern Child Health J.

[57] Meyer JP, Isaacs K, El-Shahawy O, Burlew AK, Wechsberg W (2019). Research on women with substance use disorders: Reviewing progress and developing a research and implementation roadmap. Drug Alcohol Depend.

[58] Aromataris E, Munn Z, editors. JBI Manual for Evidence Synthesis. Adelaide: JBI; 2020. Available from: https://synthesismanual.jbi.global. [Cited 2023 Jun 09].

[59] Tricco AC, Lillie E, Zarin W, O’Brien KK, Colquhoun H, Levac D (2018). PRISMA extension for scoping reviews (PRISMA-ScR): checklist and explanation The PRISMA-ScR Statement. Ann Intern Med.

[60] Health Canada. Controlled and illegal drugs. Health Canada. 2023. Available from: https://www.canada.ca/en/health-canada/services/substance-use/controlled-illegal-drugs.html. [Cited 2023 Jun 09].

[61] Page MJ, McKenzie JE, Bossuyt PM, Boutron I, Goffmann TC, Mulrow CD (2021). The PRISMA 2020 statement: an updated guideline for reporting systematic reviews. BMJ.

[62] Aventa Centre of Excellence for Women with Addictions. Pregnancy program overview. Calgary (AB): Aventa Centre of Excellence for Women with Addictions; 2023. Available from https://aventa.org/wp-content/uploads/2023/06/Pregnancy-Program-Brochure-June-2023.pdf. [Cited 2023 July 27].

[63] AMS-KFLA. Addiction Services. Kingston (ON): Addiction and Mental Health Services; 2023. Available from https://amhs-kfla.ca/programs-services/addiction-services/. [Cited 2023 July 27].

[64] Marcellus L, Nathoo T, Poole N. Harm Reduction and Pregnancy: Best and Promising Practices for Supporting Pregnant Women and New Mothers Who Use Substances. Vancouver (BC): Centre of Excellence for Women’s Health; 2016. Available from http://www.perinatalservicesbc.ca/Documents/Education/Conference/2016/Presentations2/D4iii_Marcellus.pdf. [Cited 2023 July 27].

[65] Rutman D, Hubberstey C, Van Bibber M, Poole N, Schmidt RA. Stories and Outcomes of Wraparound Programs Reaching Pregnant and Parenting Women at Risk. Victoria, BC: Nota Bene Consulting Group; 2021. Available from https://canfasd.ca/wp-content/uploads/publications/FINAL-CCE_Report_Mar-8-for-web.pdf. [Cited 2023 July 27].

[66] Provincial Perinatal Substance Use Project Team. Provincial Blueprint for a Perinatal Substance Use Continuum of Care. Vancouver (BC): BC Women’s Hospital; 2021. Available from http://www.bcwomens.ca/Professional-Resources-site/Documents/Perinatal%20Substance%20Use/PPSUP%20Blueprint%20Final%203Nov2021.pdf. [Cited 2023 July 27].

[67] Schmidt R, Wolfson L, Stinson J, Poole N, Greaves L. Mothering and Opioids: Addressing Stigma and Acting Collaboratively. Vancouver (BC): Centre of Excellence for Women’s Health; 2019. Available from https://cewh.ca/wp-content/uploads/2022/01/CEWH-03-MO-Toolkit_WEB_Update-F-1.pdf. [Cited 2023 July 27].

[68] Families in Recovery. Patient and family orientation guide. Vancouver (BC): BC Women’s Hospital and Health Care; 2021. [Cited 2023 July 27].

[69] FIR Square. Pregnant or newly parenting with substance use - goals. Vancouver (BC): BC Women’s Hospital and Health Care; 2023. Available: http://www.bcwomens.ca/our-services/pregnancy-prenatal-care/pregnancy-drugs-alcohol. [Cited 2023 July 27].

[70] Interior Health. Perinatal Counselling Services. Kelowna (BC): Interior Health; 2023. Available from: https://www.interiorhealth.ca/services/perinatal-counselling-services. [Cited 2023 July 27].

[71] Kennedy T. 'I never felt more loved or cared for': Red Deer moms-to-be healthier & drug-free thanks to EMBRACE program. Red Deer (AB): Alberta Health Services; 2022. Available from https://www.albertahealthservices.ca/news/Page17047.aspx. [Cited 2023 July 27].

[72] Smith A. ‘Everyone deserves respect’:Physicians say rooming-in program needed to support mothers struggling with substance use. Calgary (AB): Calgary Herald; 2021. Available from https://calgaryherald.com/news/local-news/detox. [Cited 2023 July 27].

[73] Zielinski S. Support expands for moms with opioid addiction and their babies. Red Deer (AB): Red Deer Advocate; 2022. Available from https://www.reddeeradvocate.com/news/support-expands-for-moms-with-opioid-addiction-and-their-babies/#. [Cited 2023 July 27].

[74] Kennedy T. EMBRACE helps newborns ease into life after opioids babies in withdrawal. Red Deer (AB): Alberta Health Services; 2019. Available from https://www.albertahealthservices.ca/news/Page15025.aspx. [Cited 2023 July 27].

[75] Urbanoski K, Joordens C, Kolla G, Milligan K (2018). Community networks of services for pregnant and parenting women with problematic substance use. PLoS One.

[76] Rutman D, Hubberstey C (2020). Cross-sectoral collaboration working with perinatal women who use substances: outcomes and lessons from HerWay Home. J Soc Work Pract Addict.

[77] Manitoba Harm Reduction Network. 2019 Manitoba Harm Reduction Gathering Report. Winnipeg (MB): Manitoba Harm Reduction Network; 2019. Available from: https://static1.squarespace.com/static/561d5888e4b0830a0f1ed08b/t/5f8f38e35d05cd5a660a6c5f/1603221740719/2019+Gathering+Report.pdf. [Cited 2023 July 27].

[78] BCAPOP. The healthy care pregnancy program. Kamloops (BC): BCAPOP; 2023. Available from https://bcapop.ca/HCPP#:~:text=The%20Healthy%20Care%20Pregnancy%20Program,are%20also%20BCAPOP%20Program%20Members. [Cited 2023 July 27].

[79] Kamloops Family Resources Society. The Tree. Kamloops (BC): Kamloops Family Resources Society; 2022. Available from https://www.kfrs.ca/. [Cited 2023 July 27].

[80] BCAPOP. Healthy care pregnancy program. Vancouver (BC): The Pregnancy Hub; 2023. Available from https://www.pregnancyhub.org/HCPP. [Cited 2023 July 28].

[81] SARA for Women. Outreach programs. Abbotsford (BC): SARA for Women; 2023. Available from: https://www.saraforwomen.ca/outreach-programs. [Cited 2023 July 28].

[82] Elizabeth Fry Society of Greater Vancouver. Program and Services. Vancouver (BC): Elizabeth Fry Society; 2023. Available from https://efry.com/we-can-help/programs-services/#recovery-transition-women. [Cited 2023 July 27].

[83] Kamloops Family Resources Society. Our Services. Kamloops (BC): Kamloops Family Resources Society; 2023. Available from: https://www.kfrs.ca/our-services/. [Cited 2023 July 27].

[84] Karis Support Society. About. Kelowna (BC): Karis Support Society; 2023. Available from https://karis-society.org/about/. [Cited 2023 July 27].

[85] Harmony House. Harmony House BC. Prince George (BC): Harmony House; 2023. Available from https://harmonyhousebc.com/. [Cited 2023 July 27].

[86] Van Bibber M, Rutman D, Hubberstey C, Poole N. A mustard seed of hope: Culturally grounded approaches within wraparound care for pregnant and parenting women dealing with substance use and trauma. Victoria (BC): Nota Bene Consulting Group; 2022. Available from https://cewh.ca/wp-content/uploads/2023/03/Final-MSOH-booklet-web-quality-version-2.pdf. [Cited 2023 July 27].

[87] Atira Women’s Society. The Maxxine Wright Community Health Centre. Surrey (BC): Atira Women’s Society; 2023. Available from: https://atira.bc.ca/what-we-do/program/maxxine-wright-community-health-centre/. [Cited 2023 July 28].

[88] FIR Square. Pregnant or newly parenting with substance use - services. Vancouver (BC): BC Women’s Hospital and Health Care; 2023. Available http://www.bcwomens.ca/our-services/pregnancy-prenatal-care/pregnancy-drugs-alcohol#Services. [Cited 2023 July 27].

[89] Vancouver Coastal Health. Sheway fact sheet. Vancouver (BC): Vancouver Coastal Health; 2018. Available from https://www.vch.ca/sites/default/files/import/documents/sheway-fact-sheet.pdf. [Cited 2023 July 28].

[90] Hubberstey C, Rutman D, Van Bibber M, Poole N (2022). Wraparound programmes for pregnant and parenting women with substance use concerns in Canada: Partnerships are essential. Health Soc Care Community.

[91] Kashak, D. An Overview of Issues, Impacts and Services for Women who are Using Substances and are Pregnant or Parenting within the City of Thunder Bay: Literature Review & Environmental Scan of Programs in Thunder Bay. 2016.

[92] Pathways Vancouver. Sheway - For pregnant women and new mothers with drug and alcohol issues. Vancouver (BC): Pathways Vancouver Community Service Directory; 2023. Available from https://vancouver.pathwaysbc.ca/programs/444. [Cited 2023 July 27].

[93] Vancouver Coastal Health. Pregnancy Outreach Program at Sheway. Vancouver (BC): Vancouver Coastal Health; 2023. Available from: https://www.vch.ca/en/location-service/pregnancy-outreach-program-sheway. [Cited 2023 July 27].

[94] Vancouver Coastal Health. Sheway Pregnancy Outreach Program. Vancouver (BC): Vancouver Coastal Health; 2023. Available from https://www.vch.ca/en/service/sheway-pregnancy-outreach-program. [Cited 2023 July 28].

[95] L’Espérance N, Bertrand K, Perreault M. L’intervention auprès des femmes enceintes et mères consommatrices de psychotropes. Drogue, santé et société. 2018;14(2):90- 108.

[96] Gartner K, Elliott K, Smith M, Pearson H, Hunt G, Martin RE (2018). "People in regular society don't think you can be a good mother and have a substance use problem": Participatory action research with women with substance use in pregnancy. Can Fam Physician.

[97] Ritland L, Jongbloed K, Mazzuca A (2020). Culturally Safe, Strengths-Based Parenting Programs Supporting Indigenous Families Impacted by Substance Use—a Scoping Review. Int J Ment Health Addiction.

[98] Rutman D, Hubberstey C, Poole N, Schmidt RA, Van Bibber M (2020). Multi-service prevention programs for pregnant and parenting women with substance use and multiple vulnerabilities: Program structure and clients' perspectives on wraparound programming. BMC Pregnancy Childbirth.

[99] Hubberstey C, Rutman D, Schmidt RA, Van Bibber M, Poole N (2019). Multi-service programs for pregnant and parenting women with substance use concerns: women's perspectives on why they seek help and their significant changes. Int J Environ Res Public Health.

[100] Island Health. HerWay Home (HWH). Victoria (BC): Island Health; 2023. Available from https://www.islandhealth.ca/learn-about-health/pregnancy-birth-babies/herway-home-hwh. [Cited 2023 July 27].

[101] Hubberstey C, Rutman D (2020). HerWay Home Program for Pregnant and Parenting Women Using Substances: A Brief Social Return on Investment Analysis. Can J Addict.

[102] Fernwood Neighbourhood Resource Group. Family programs. Victoria (BC): Fernwood Neighbourhood Resource Group; 2021. Available from https://fernwoodnrg.ca/fernwood-nrg-programs/family-programs/. [Cited 2023 July 27].

[103] Healthy Families Healthy Futures. Parent Child Assistance Program. Westlock (AB): Healthy Families Healthy Futures; 2023. Available from https://www.hfalberta.com/parent-child-assistance-program#:~:text=PCAP%20serves%20women%20who%20are,are%20unable%20to%20achieve%20sobriety. [Cited 2023 July 27].

[104] McMan Calgary. Parent-child assistance program (P-CAP). Calgary (AB): McMan Calgary; 2023. Available from https://mcmancalgary.ca/pcap/. [Cited 2023 July 28].

[105] Recovery Access Alberta. Aventa Centre of Excellence for Women with Addictions. Calgary (AB): Recovery Access Alberta; 2023. Available from https://recoveryaccessalberta.ca/service/aventa-centre-of-excellence-for-women-with-addictions/. [Cited 2023 July 27].

[106] CATIE. The H.E.R. Pregnancy Program. Toronto (ON): CATIE; 2018. Available from https://www.catie.ca/programming-connection/the-her-pregnancy-program#:~:text=child(ren).-,The%20H.E.R.,The%20H.E.R. [Cited 2023 July 27].

[107] Alberta Health Services. Health for two. Edmonton (AB): Alberta Health Services; 2023. Available from https://www.albertahealthservices.ca/findhealth/Service.aspx?id=5807. [Cited 2023 July 28].

[108] Turning Point Society. Turning Point - Women’s Program. Red Deer (AB): Turning Point Society; 2022. Available from https://turningpoint-ca.org/womens-program/. [Cited 2023 July 27].

[109] Sanctum Care Group. PORT (Prenatal outreach and resource team). Saskatoon (SK): Sanctum Care Group; 2023. Available from https://sanctumcaregroup.com/programs/port-prenatal-outreach-and-resource-team#:~:text=PORT%20will%20function%20as%20a,%2C%20social%20(peer%20navigators%2C%20and. [Cited 2023 July 27].

[110] HelpSeeker. SWAP - Residential Support (Raising Hope - Moving Families Forward). Calgary (AB): Help Seeker; 2020. Available from https://search.helpseeker.org/canada/saskatchewan/regina/swap-residential-support-raising-hope-moving-families-forward. [Cited 2023 July 27].

[111] Healthy Child Manitoba. Treatment and Care for Pregnant Women who use alcohol and/or other drugs. Winnipeg (MB): Healthy Child Manitoba; 2016. Available from: https://www.gov.mb.ca/fs/fasd/pubs/treatmentcare_pregnantwomen_more.pdf. [Cited 2023 November 15].

[112] Province of Manitoba. InSight Mentoring Program. Winnipeg (MB): Province of Manitoba; 2023. Available from: https://www.gov.mb.ca/fs/fasd/insight.html. [Cited 2023 July 27].

[113] Province of Manitoba. Insight Mentoring Program for Service Providers. Winnipeg (MB): Province of Manitoba; 2023. [Cited 2023 July 27]. Available from https://www.gov.mb.ca/fs/fasd/pubs/insightsp_en.pdf.

[114] Province of Manitoba. Insight program: support for pregnant women and new mothers who use substances. Winnipeg (MB): Province of Manitoba; 2023. Available from: https://www.gov.mb.ca/fs/fasd/pubs/insightclient_en.pdf. [Cited 2023 July 28].

[115] Hamilton Niagara Haldimand Brant Local Health Integration Network. Salvation Army - Hamilton - Grace Haven Young Parent Resource Centre. Hamilton (ON): Hamilton Niagara Haldimand Brant Local Health Integration Network; 2023. Available from: https://www.hnhbhealthline.ca/displayservice.aspx?id=179999. [Cited 2023 July 28].

[116] Kingston Community Health Centres. Thrive. Kingston (ON): Kingston Community Health Centre; 2023. Available from https://kchc.ca/weller-avenue/thrive/. [Cited 2023 July 27].

[117] Bueckart J, Gaudet L., McGee A, McLellan A, Sellers L, Willows M. The ORACLE Collaborative Pathway - For Pregnant People Who Use Substances. Mississauga (ON): Canadian Collaborative Mental Health Initiative; 2017. Available from http://www.shared-care.ca/files/The_Oracle_Collaborative_Pathway_Condensed.pdf. [Cited 2023 July 28].

[118] Monarch Recovery Services. Pregnancy/Parenting Outreach Program (PPOP). Sudbury (ON): Monarch Recovery Services; 2022. Available from https://monarchrecoveryservices.ca/i-want-help/making-changes/ppop-pregnancyparenting-outreach-program#:~:text=This%20program%20is%20a%20harm,develop%20a%20variety%20of%20skills. [Cited 2023 July 28].

[119] Mothercraft. Breaking the cycle. Toronto (ON): Canadian Mothercraft Society; 2016. Available from https://www.mothercraft.ca/index.php?q=ei-btc. [Cited 2023 July 27].

[120] Mothercraft. Evaluation. Toronto (ON): Canadian Mothercraft Society; 2016. Available from https://www.mothercraft.ca/index.php?q=breaking-the-cycle-evaluation. [Cited 2023 July 27].

[121] Andrews NCZ, Motz M, Pepler DJ, Jeong JJ, Khoury J (2018). Engaging mothers with substance use issues and their children in early intervention: Understanding use of service and outcomes. Child Abuse Negl.

[122] Ramirez S. Bridges to Moms. Toronto (ON): Alliance for Healthier Communities; 2023. Available from https://www.allianceon.org/Bridges-Moms#:~:text=The%20program%20is%20led%20by,programs%20and%20child%20development%20services. [Cited 2023 July 28].

[123] Toronto Public Health. Toronto Public Health - Homelessness at Risk Prenatal (HARP). Toronto (ON): Toronto Public Health; 2023. Available fromhttps://www.cpha.ca/sites/default/files/assets/progs/soc-detrmnts/frontline/harp-risk.pdf. [Cited 2023 July 27].

[124] Toronto Central Local Health Integrated Network. Toronto Centre for Substance Use in Pregnancy. Toronto (ON): Toronto Public Health; 2023. Available from: https://www.torontocentralhealthline.ca/displayservice.aspx?id=161649. [Cited 2023 July 27].

[125] Mothercraft. Programs and services. Toronto (ON): Canadian Mothercraft Society; 2016. Available from https://www.mothercraft.ca/index.php?q=breaking-the-cycle-programs-and-services. [Cited 2023 July 28].

[126] Gander S. Advocacy and Empowerment of Women who Use Substances during Pregnancy: Reports from the Parent Child Assistance Program (PCAP) in Saint John, NB. Saint John (NB): NB Social Pediatrics; 2022. Available from https://static1.squarespace.com › static › PP+-+D... [Cited 2023 July 27].

[127] IWK Health Centre. Having a baby. Halifax (NS): IWK Health Centre; 2023. Available from https://www.iwk.nshealth.ca/women-and-newborns-health/services/having-a-baby. [Cited 2023 July 28].

[128] Nathoo T, Poole N, Wolfson L, Schmidt R, Hemsing N, Gelb K. Doorways to Conversation: Brief Intervention on Substance Use with Girls and Women. Vancouver, BC: Centre of Excellence for Women’s Health; 2018. Available from: https://cewh.ca/wp-content/uploads/2018/06/Doorways_ENGLISH_July-18-2018_online-version.pdf. [Cited 2023 July 27].

[129] Parkdale Queen West Community Health Centre. Case Management for At-risk Pregnant and Parenting Women. Toronto (ON): Parkdale Queen West Community Health Centre; 2023. Available from: https://pqwchc.org/programs-services/community-services-and-programs/pregnancy-and-parenting-programs/case-management-for-at-risk-pregnant-and-parenting-women/. [Cited 2023 July 28].

[130] L'Espérance N, Bertrand K, Perreault M (2017). Cross-training to work better together with women in Quebec who use substances: care providers' perceptions. Health Soc Care Community.

[131] Milligan K, Tarasoff LA, Ingram V. Integrated treatment programs for pregnant and parenting women in Ontario: Models, processes, and outcomes. Toronto (ON): Addictions and Mental Health Ontario; 2018. Available from: https://amho.ca/wp-content/uploads/IC6-Integrated-Treatment-Programs-for-Pregnant-and-Parenting-Women-in-Ontario.pdf. [Cited 2023 July 27].

[132] Tarasoff LA, Milligan K, Le TL, Usher AM, Urbanoski K (2018). Integrated treatment programs for pregnant and parenting women with problematic substance use: Service descriptions and client perceptions of care. J Subst Abuse Treat.

[133] Cidro J, Doenmez C, Sinclair S (2021). Putting them on a strong spiritual path: Indigenous doulas responding to the needs of Indigenous mothers and communities. Int J Equity Health.

[134] Olding M, Cook A, Austin T, Boyd J (2022). "They went down that road, and they get it": a qualitative study of peer support worker roles within perinatal substance use programs. J Subst Abuse Treat.

[135] Milligan K, Usher AM, Urbanoski KA (2017). Supporting pregnant and parenting women with substance-related problems by addressing emotion regulation and executive function needs. Addict Res Theory.

[136] Doberstein C, Insite in Vancouver: North America’s First Supervised Injection Site. In: Lindquist E, Howlett M, Skogstad G, Tellier G, t’Hart P, editors. Policy Success in Canada: Cases, Lessons, Challenges. Oxford (GB): Oxford Academic; 2022. 10.1093/oso/9780192897046.003.0004 [Cited 2023 July 14].

[137] Belzak L, Halverson J (2018). The opioid crisis in Canada: a national perspective. Health Promot Chronic Dis Prev Can.

[138] Vancouver Coastal Health. Insite - Vancouver Coastal Health. Vancouver (BC): Vancouver Coastal Health; 2023. Available from: www.vch.ca/en/location/insite. [Cited 2023 July 17].

[139] Campbell L, Boyd N, Culbert L (2009). A thousand dreams: Vancouver's Downtown Eastside and the fight for its future.

[140] City of Vancouver. Vancouver’s Approach to the Overdose Crisis. Vancouver (BC): City of Vancouver; 2019. Available from vancouver.ca/people-programs/drugs.aspx. . [cited 2023 July 26]

[141] Macfarlane E, Johnstone R (2021). Equality rights, abortion access, and New Brunswick's regulation 84–20. UNBLJ.

[142] Action Canada for Sexual Health and Rights. Pre-Budget Consultations 2022: Recommendations for Strengthening SRHR in Canada. Ottawa (ON): Action Canada for Sexual Health and Rights; 2022. Available from www.actioncanadashr.org/news/2022-03-09-pre-budget-consultations-2022-recommendations-strengthening-srhr-canada. [Cited 2023 July 17].

[143] Pauktuutit Inuit Women of Canada. Access, respect, consent: Inuit women and reproductive healthcare services in 2019. Ottawa: Pauktuutit; 2020. Available from: https://pauktuutit.ca/wp-content/uploads/Pauktuutit_Reproductive-Health_English_04.pdf. [Cited 2023 November 16].

[144] Government of Yukon. Substance use health emergency. Whitehorse (YK): Government of Yukon; 2022. Available from: https://yukon.ca/en/substance-use-health-emergency. [Cited 2023 July 30].

[145] Nelson E (2016). Autonomy, equality, and access to sexual and reproductive health care. Alta L Rev.

[146] Frontline AIDS. Advancing the sexual and reproductive health and rights of women who use drugs. East Sussex: Frontline AIDS; 2020. Available from: https://frontlineaids.org/wp-content/uploads/2020/02/Guide-for-harm-reduction-programmes-FINAL-24Feb-WEB.pdf. [Cited 2023 November 15].

[147] Yee J, Apale AN, Deleary M (2011). Sexual and reproductive health, rights, and realities and access to services for first nations, inuit, and métis in Canada. J Obstet Gynaecol Can.

[148] Moazen-Zadeh E, Karamouzian M, Kia H, Salway T, Ferlatte O, Knight R (2019). A call for action on overdose among LGBTQ people in North America. Lancet Psychiatry.

[149] Rojas JI, Leckie R, Hawks EM, Holster J, del Carmen Trapp M, Ostermeyer BK (2019). Compounded Stigma in LGBTQ+ People: a framework for understanding the relationship between substance use disorders, mental illness, trauma, and sexual minority status. Psychiatr Ann.

[150] Lavalley J, Kastor S, Valleriani J, McNeil R (2018). Reconciliation and Canada's overdose crisis: responding to the needs of Indigenous Peoples. CMAJ.

[151] Minister of Indigenous Services. Annual report to Parliament 2020. Ottawa: Government of Canada; 2020. Available from https://www.sac-isc.gc.ca/eng/1602010609492/1602010631711 [Cited 2023 July 27].

[152] Friedman SR, Mateu-Gelabert P, Nikolopoulos GK, Cerdá M, Rossi D, Jordan AE, Townsend T, Khan MR, Perlman DC (2021). Big Events theory and measures may help explain emerging long-term effects of current crises. Glob Public Health..

