# Supplementary material for: Programs and practices that support pregnant people who use drugs’ access to sexual and reproductive health care in Canada: a scoping review
Source: BMC Pregnancy Childbirth. 2024 Jan 22;24:72. doi: 10.1186/s12884-023-06225-w (PMC10804510; doi:10.1186/s12884-023-06225-w)
Supplement: Supplementary file 1 — Additional file 1. Sustainable Development Goals relevant to pregnant people who use drugs. [file 12884_2023_6225_MOESM1_ESM.docx]

**Additional File 1. Sustainable Development Goals relevant to pregnant people who use drugs**

| **Goal 3 -** Ensure healthy lives and promote well-being for all at all ages | |
| --- | --- |
| **Targets** |  |
| 3.1 | By 2030, reduce the global maternal mortality ratio to less than 70 per 100,000 live births |
| 3.2 | By 2030, end preventable deaths of newborns and children under 5 years of age, with all countries aiming to reduce neonatal mortality to at least as low as 12 per 1,000 live births and under-5 mortality to at least as low as 25 per 1,000 live births |
| 3.5 | Strengthen the prevention and treatment of substance abuse, including narcotic drug abuse and harmful use of alcohol |
| 3.7 | By 2030, ensure universal access to sexual and reproductive health-care services, including for family planning, information and education, and the integration of reproductive health into national strategies and programmes |
| 3.8 | Achieve universal health coverage, including financial risk protection, access to quality essential health-care services and access to safe, effective, quality and affordable essential medicines and vaccines for all |
| **Goal 5** - Achieve gender equality and empower all women and girls | |
| 5.6 | Ensure universal access to sexual and reproductive health and reproductive rights as agreed in accordance with the Programme of Action of the International Conference on Population and Development and the Beijing Platform for Action and the outcome documents of their review conferences |
| 5.c | Adopt and strengthen sound policies and enforceable legislation for the promotion of gender equality and the empowerment of all women and girls at all levels |
| **Goal 11 -** Make cities and human settlements inclusive, safe, resilient and sustainable | |
| 11.2 | By 2030, provide access to safe, affordable, accessible and sustainable transport systems for all, improving road safety, notably by expanding public transport, with special attention to the needs of those in vulnerable situations, women, children, persons with disabilities and older persons |

File 1. An outline of the key Sustainable Development Goals that are most relevant to pregnant people who use drugs
