# Supplementary material for: Programs and practices that support pregnant people who use drugs’ access to sexual and reproductive health care in Canada: a scoping review
Source: BMC Pregnancy Childbirth. 2024 Jan 22;24:72. doi: 10.1186/s12884-023-06225-w (PMC10804510; doi:10.1186/s12884-023-06225-w)
Supplement: Supplementary file 2 — Additional file 2. Search strategy. [file 12884_2023_6225_MOESM2_ESM.docx]

# Additional File 2: Search strategy

## MEDLINE (Ovid)

Search conducted on April 3, 2023 in keyword field

| **Search** | **Query** | **Records retrieved** |
| --- | --- | --- |
| #1 | (pregnan* or prenatal or antenatal or perinatal or antepartum or gestat*).mp | 1,254,069 |
| #2 | ("illicit drug*" or "illegal drug*").mp | 24281 |
| #3 | ("bath salts" or cocaine or ketamine or "magic mushroom*" or methamphetamine or PCP or "angel dust" or codeine or benzodiazepine* or fentanyl or heroin or LSD or hallucinogen* or MDMA or ecstasy or opioid* or opiate* or salvia).mp | 337266 |
| #4 | ("drug* addict*" or "drug user*" or "drug use" or "using drug*" or "drug abus*" or "drug misuse" or "substance abuse" or "substance misuse" or "substance use") | 177162 |
| #5 | 2 or 3 or 4 | 478605 |
| #6 | 1 and 5 | 22655 |
| #7 | (Canad* OR "British Columbia" OR "Colombie Britannique" OR Alberta* OR Saskatchewan OR Manitoba* OR Ontario OR Quebec OR "Nouveau Brunswick" OR "New Brunswick" OR "Nova Scotia" OR "Nouvelle Ecosse" OR "Prince Edward Island" OR Newfoundland OR Labrador OR Nunavut OR NWT OR "Northwest Territories" OR Yukon OR Nunavik OR Inuvialuit) | 272259 |
| #8 | 6 and 7 | 493 |
| Limited to English and French articles | |  |
